# Convergent evolution of a labile nutritional symbiosis in ants

**DOI:** 10.1038/s41396-022-01256-1

**Published:** 2022-06-14

**Authors:** Raphaella Jackson, David Monnin, Patapios A. Patapiou, Gemma Golding, Heikki Helanterä, Jan Oettler, Jürgen Heinze, Yannick Wurm, Chloe K. Economou, Michel Chapuisat, Lee M. Henry

**Affiliations:** 1 School of Biological and Behavioural Sciences, Queen Mary University of London, London, E1 4NS UK; 2 Department of Pathobiology and Population Sciences, Royal Veterinary College, Hatfield, AL9 7TA UK; 3 Ecology and Genetics Research Unit, University of Oulu, Oulu, 90014 Finland; 4 Tvärminne Zoological Station, University of Helsinki, Hanko, Finland; 5 Zoology/Evolutionary Biology, University of Regensburg, Regensburg, 93040 Germany; 6 Alan Turing Institute, London, NW1 2DB UK; 7 Department of Ecology and Evolution, University of Lausanne, 1015 Lausanne, Switzerland

**Keywords:** Molecular evolution, Microbial ecology, Comparative genomics

## Abstract

Ants are among the most successful organisms on Earth. It has been suggested that forming symbioses with nutrient-supplementing microbes may have contributed to their success, by allowing ants to invade otherwise inaccessible niches. However, it is unclear whether ants have evolved symbioses repeatedly to overcome the same nutrient limitations. Here, we address this question by comparing the independently evolved symbioses in *Camponotus*, *Plagiolepis*, *Formica* and *Cardiocondyla* ants. Our analysis reveals the only metabolic function consistently retained in all of the symbiont genomes is the capacity to synthesise tyrosine. We also show that in certain multi-queen lineages that have co-diversified with their symbiont for millions of years, only a fraction of queens carry the symbiont, suggesting ants differ in their colony-level reliance on symbiont-derived resources. Our results imply that symbioses can arise to solve common problems, but hosts may differ in their dependence on symbionts, highlighting the evolutionary forces influencing the persistence of long-term endosymbiotic mutualisms.

## Introduction

Ants are among the most ecologically dominant organisms in terrestrial ecosystems, and part of their success lies in their ability to occupy a wide range of habitats. It has been suggested that acquiring nutrient-provisioning symbionts may have allowed certain ant lineages to survive in nutrient imbalanced habitats. For example, gut-associated bacteria are thought to have enabled transitions to arboreal lifestyles in several ant lineages by relaxing their need for nitrogen and allowing them to feed predominantly on plant-derived resources, such as extrafloral nectaries and insect honeydew [1–3]. Symbiont acquisitions may therefore represent key adaptations that have allowed ants to significantly expand the ecological niches in which they can forage and complete development, thereby contributing to their widespread ecological success.

At least four ant lineages have evolved symbioses where the microbes are housed within specialised cells called bacteriocytes that surround the midgut. Bacteriocytes are a common feature of ancient nutritional symbioses, where symbionts are essential for host development and strictly vertically transmitted through host generations [4]. The most well-studied bacteriocyte-associated symbiont in ants is *Blochmannia*, the obligate symbiont of *Camponotus* carpenter ants (Formicidae: Formicinae). *Blochmannia* provides its host with essential amino acids that can improve brood production, especially when proteins are scarce [1]. *Blochmannia* is also thought to aid hosts in nitrogen recycling and synthesises the aromatic amino acid tyrosine, which is essential for insect cuticle synthesis and other physiological processes such as melanisation and sclerotisation [5–7]. The symbiont of *Cardiocondyla obscurior* (Formicidae: Myrmicinae), *Candidatus* Westeberhardia cardiocondylae, hereafter *Westeberhardia*, despite having a highly reduced genome, has also retained the capacity to synthesise tyrosine through a shared metabolic pathway with its ant host [8]. Two additional ant genera, *Formica* and *Plagiolepis*, are also known to harbour symbionts within bacteriocytes surrounding the midgut, suggesting they also play a role in provisioning nutrients for their hosts [9–11]. However, the functional role of the symbionts in *Formica* and *Plagiolepis* is currently unknown.

While the acquisition of nutrient provisioning symbionts has allowed insects to repeatedly invade nutrient imbalanced niches, such as plant sap and blood feeding [12], it is less clear why these relationships evolve in predominantly omnivorous insects such as ants. Stable isotope analyses of *Camponotus* and *Plagiolepis* have suggested ants in these genera feed predominantly on plant-based resources [13]. Similarly, both *Cardiocondyla* and *Formica* commonly feed on honeydew and nectar [14, 15], and symbiont-carrying *Formica* species have been shown to occupy a lower trophic position than asymbiotic species [16]. This suggests that symbiont acquisitions may have facilitated convergent shift from omnivory to more plant-based diets in each of these ant lineages. However, it is currently unclear whether the symbioses have evolved to supplement the same vital nutrients limiting in their hosts’ diets. This has limited our understanding of the metabolic challenges facing omnivorous insects, and how nutritional symbioses evolve to overcome them.

The aim of this study is to determine whether the four bacteriocyte-associated symbioses in ants represent ancient nutritional mutualisms that have evolved to serve similar functions for their hosts. We first characterise the genomes of the symbionts in *Formica* and *Plagiolepis*, and several new strains of *Westeberhardia* from phylogenetically divergent *Cardiocondyla* lineages. Using a comparative approach, we asked whether the symbionts from all four ant lineages have retained metabolic pathways in their highly reduced genomes that suggest they serve similar nutrient-provisioning roles for their hosts. We then investigated the phylogenetic and intracolony distributions of symbionts in diverse *Formica* and *Cardiocondyla* species to determine the origins of each symbiosis and its prevalence across species and castes. This survey reveals that despite retaining their symbiont for millions of years, in many ant lineages that maintain multi-queen (polygynous) colonies, only a fraction of queens carry the symbiont, suggesting species differ in their dependence on symbiont-derived nutrients at the colony level. We present evidence that suggests species differences in symbiont retention are not correlated with changes in symbiont functionality and discuss how ant feeding ecology, sociality and cost-benefit trade-offs may impact dependence on nutritional symbioses.

## Results and discussion

### Genome characteristics of ancient obligate symbionts

We first tested the hypothesis that each of the ant lineages sequenced in our study (*Cardiocondyla*, *Formica*, and *Plagiolepis*) hosts its own ancient strictly vertically transmitted symbiont that have co-speciated with its host, which has been shown previously in the *Camponotus*- *Blochmannia* symbiosis [17]. To address this aim, we compared the genomes of symbionts from 13 species of ants, 8 from our study combined with 5 previously published genomes, representing four independently evolved symbioses. This includes symbionts from three *Formica*, two *Plagiolepis*, and an additional three *Cardiocondyla* species that we sequenced, in addition to four previously published genomes from *Blochmannia*, the obligate symbiont of *Camponotus* ants, and the one pre-existing *Westeberhardia* genome from *Cardiocondyla obscurior* [8, 18–21].

**Fig. 1:**
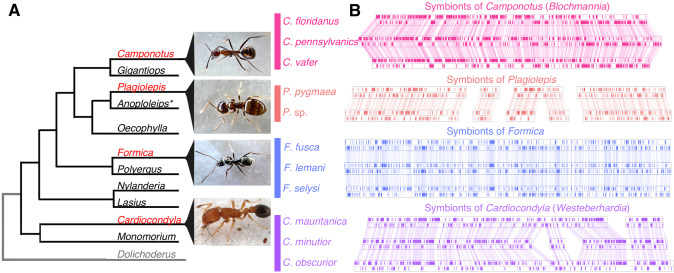
Structural stability of ant symbiont genomes. **A** Ant lineages known to host bacteriocyte-associated symbionts (red font) and lineages not known to (black font), based on [91]. Outgroup (grey font) not examined in this study. **B** Visualisation of symbiont genomes showing conservation of gene order in the symbionts of ant species that belong to the same genus. Blocks show the locations of single copy orthologs in the symbiont genome, lines connect shared single copy orthologs between genomes. All genomes and annotations were generated in this study except the *Blochmannia* symbionts and the *Westeberhardia* strain from *C. obscurior* [8, 18–21]. *Evidence of symbionts were detected in embryos of *Anoplolepis* [91] but it is unclear if they are localised in bacteriocytes in larvae and adults.

We found the gene order of single copy orthologs in symbionts is highly conserved in ant species belonging to the same genus (Fig. 1). This type of structural stability of genomes is typically found in symbionts that have been strictly vertically transmitted within a matriline [22] and has been documented in the obligate symbionts of whiteflies, psyllids, cockroaches, and aphids [23–26]. In contrast, genome structure differed substantially between symbionts from different ant genera (Fig. 1, Fig. S1). We also find that the host and symbiont phylogenies are in general concordance in *Cardiocondyla* (TreeMap: *p* = 0.00100 CI_95%_ = [0.00000, 0.00424]), and in *Formica* the topologies suggest co-segregation, although there were too few nodes to confirm this statistically (Fig. S2). Together, this strongly suggests the symbioses in all four ant lineages are independently acquired ancient associations that have co-speciated with their hosts.

**Fig. 2:**
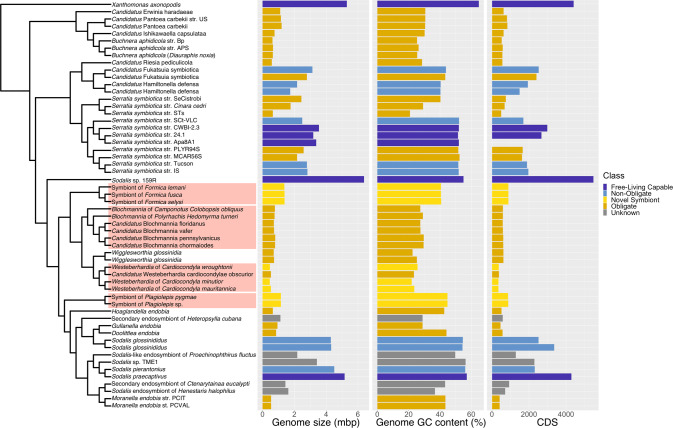
Phylogenetic origins of the bacteriocyte-associated symbionts of ants. A pruned phylogeny of gammaproteobacterial endosymbionts based on Fig. S8. The phylogeny is based on a dayhoff6 recoded amino acid alignment of 72 genes analysed using phylobayes. Bar plots represent the size (in Mbp) and GC content of symbiont genomes. Bars are colour coded to represent hypothesised relationships between symbionts and hosts. Species names highlighted in red in the phylogeny indicate the four bacteriocyte-associated symbionts of ants. Genomes sequenced and assembled for this paper are referenced as ‘novel symbiont’ lineages. Full phylogenies with node support and branch lengths are available as Fig. S8 and Fig. S9, respectively.

In addition, our phylogenetic analysis reveals that all four symbiont lineages originate from a single clade, the *Sodalis*-allied bacteria (Fig. 2). This demonstrates that ant lineages that host bacteriocytes-associated symbionts have convergently acquired related bacteria, which differs from previous findings based on limited taxa and genes [27]. All of the symbionts have evidence of advanced genome reduction, which is characterized by reduced genome size, GC content, and number of coding sequences, similar to other ancient obligate symbionts of insects [4]. The three strains of *Westeberhardia* we analysed have extremely small (0.45–0.53 Mb) GC depleted genomes (22–26%) that are similar to the figures reported for the strain in *Cardiocondyla obscurior* [8]; confirming that they have some of the smallest genomes of any known gammaproteobacterial endosymbiont (Fig. 2). By comparison, the symbionts in *Formica* and *Plagiolepis* have genomes around twice the size (1.37–1.38 Mb) and GC content (~41%) of *Westeberhardia* (Fig. 2) raising the possibility that they are in an earlier stage of genome reduction than both *Westeberhardia* and *Blochmannia*. The *Formica* and *Plagiolepis* symbionts have a similar size, GC range, and number of coding sequences as known obligate symbionts such as *Candidatus* Doolittlea endobia [28], and several *Serratia symbiotica* lineages that are co-obligate symbionts in aphids [29].

### Bacteriocyte-associated endosymbionts

Using fluorescent in situ hybridisation, we determine whether the *Sodalis*-allied symbionts we sequenced are localised in bacteriocytes to confirm they are the associations first observed by Lillienstern and Jungen in the early 1900’s [10, 11].

**Fig. 3:**
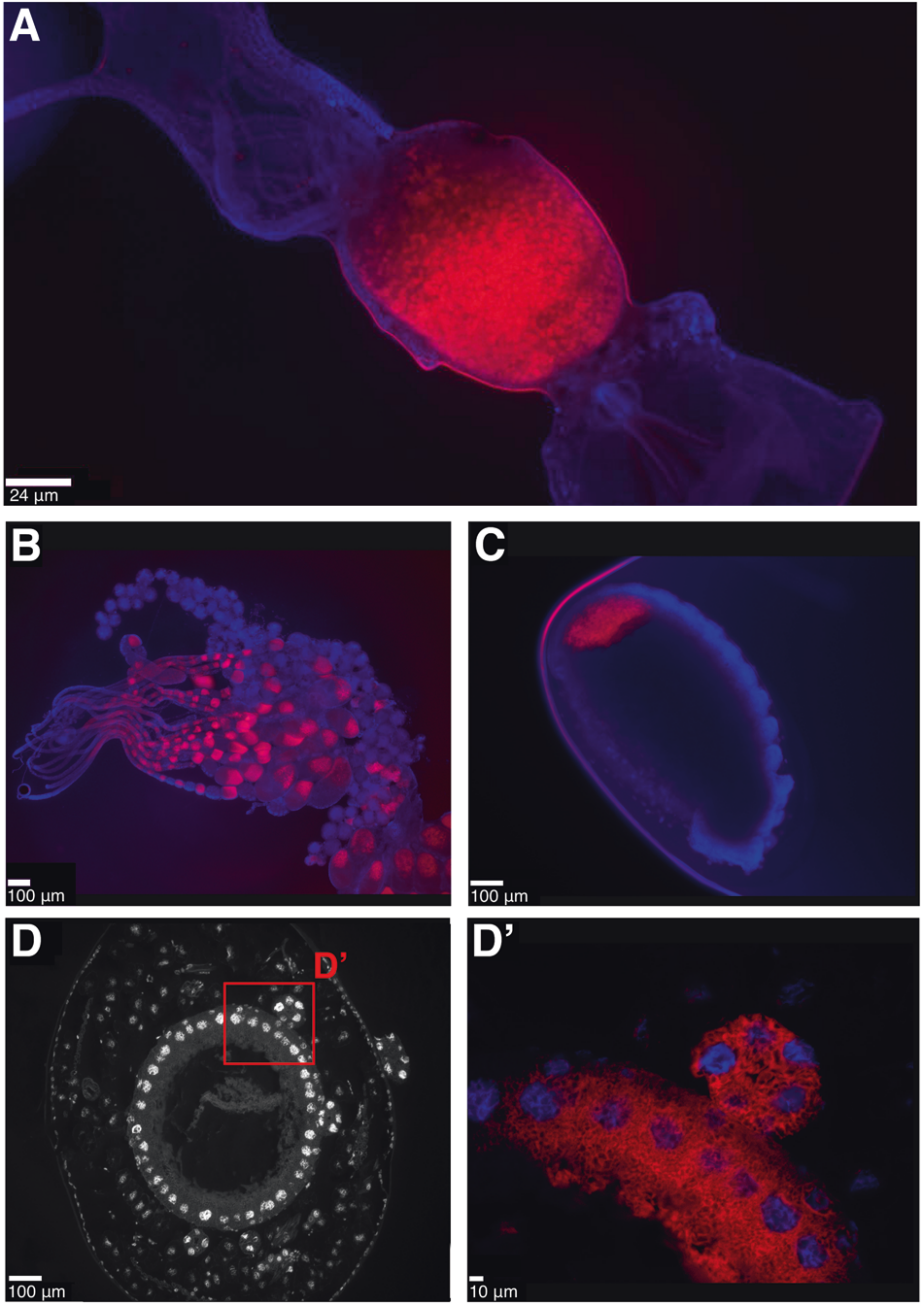
Anatomical localisation of symbiont in *Formica* ants. Fluorescent in situ hybridisation (FISH) generated images showing the localisation of symbionts in *Formica* ants. **A**–**C** Whole mount FISH of *Formica fusca*: queen gut (**A**, crop and proventriculus on the right, midgut in the middle, hindgut and Malpighian tubules on the left), ovaries (**B**) and egg (**C**). DAPI staining of host tissue in blue, symbiont stained in red. D–D’. FISH on transverse cytological sections of *Formica cinerea* larva midgut. DAPI staining only, showing host nuclei of bacteriocytes in a single layer surrounding the midgut (**D**), and a magnified region highlighting symbionts in red localised within bacteriocytes and in a bacteriome (D’). A FISH image of the symbiont-free midgut of a *Formica lemani* queen is available as Fig. S11.

Consistent with Lilienstern’s findings [11], we found the *Sodalis* symbiont in *Formica* ants is distributed in bacteriocytes surrounding the midgut in adult queens (Fig. 3A). The symbionts are also found in eggs and ovaries of adult queens, indicating they are vertically transmitted from queens to offspring (Fig. 3B–C). Sectioning of *F. cinerea* larvae shows the bacteriocytes to be arranged in a single layer of cells surrounding the midgut, as well as in clusters of bacteriocytes closely situated to the midgut (Fig. 3D–D’). In adult *Plagiolepis* queens, the symbiont was not present in bacteriocytes around the midgut, suggesting the symbiont may play a more substantive role in larval development or pupation and then migrates to the ovaries prior to or during metamorphosis. Apart from that, the localisation of the symbiont in *Plagiolepis* was the same as in *Formica* – symbionts in larval midgut bacteriocytes, ovaries and eggs (Fig. S3) – supporting Jungen’s cytological findings [10]. Bacteriocytes are also found surrounding the midgut in *Camponotus* and *Cardiocondyla* ants [8, 30, 31] indicating the symbionts are localised in a similar manner in all four ant lineages.

### Conservation of metabolic functions in ant endosymbionts

Despite on-going genome reduction, obligate symbionts of insects typically retain gene networks required for maintaining the symbiosis with their host, such as pathways for synthesising essential nutrients. This has resulted in the symbionts of sap- and blood-feeding insects converging on genomes that have retained the same sets of metabolic pathways – to synthesise essential nutrients missing in their hosts’ diets [32, 33]. Here we compare the metabolic pathways retained in the reduced genomes of the four bacteriocyte-associated symbionts of ants to test the hypothesis that have been acquired to perform similar functions. For this, we assess whether they have consistently retained metabolic pathways to synthesise the same key nutrients. Two major patterns stand out.

The first major pattern we find is that the four ant symbionts have all retained the shikimate pathway, which produces chorismate, along with most of the steps necessary to produce tyrosine from this precursor (Tables 1 and S2). Both the symbiont of *Formica* and *Westeberhardia* each lack one of the genes required to produce tyrosine. However, in *Westeberhardia* it is believed the host encodes the missing gene, supplying the enzyme to fulfil the final step of the pathway [8]. Intriguingly, we find that this gene is also present in the *Formica* ant genomes (Fig. S4). In addition, all symbionts except *Westeberhardia* can produce phenylalanine which is a precursor that can be converted to tyrosine by their hosts [5, 34, 35]. Tyrosine is important for insect development as it is used to produce L-DOPA, which is a key component of insect cuticles [5]. Tyrosine is also a precursor for melanin synthesis, which is important in protection against pathogens, and plays a fundamental role in neurotransmitters and hormone production [36, 37]. In several species of ants, weevils, and other beetles, symbionts are believed to provision hosts with tyrosine, and it has been shown experimentally in several of these species that removal or inhibition of their symbionts causes cuticle development to suffer [38–47]. A thicker cuticle has been shown to help symbiont-carrying grain beetles resist desiccation [43], and defend against natural enemies [48]. However, female reproduction is delayed at higher humidity, suggesting a metabolic cost to carrying their *Bacteroidetes* symbiont. Tyrosine provisioning is also the likely function of *Westeberhardia* in *Cardiocondyla* ants, as this is one of the few nutrient pathways retained in this symbiont. Our analysis confirms the shikimate pathway, and the symbiont portions of the tyrosine pathway, have been retained in *Westeberhardia* from three phylogenetically diverse *Cardiocondyla* lineages, providing additional support for this hypothesis. In addition to tyrosine, most of the symbionts have retained the capacity to produce vitamin B9 (tetrahydrofolate) and all can perform the single step conversions necessary to produce alanine and glycine. However, our gene enrichment analysis indicates that tyrosine, and the associated chorismate biosynthetic process, are the only enriched vitamin or amino acid pathways that are shared by all of the symbiont genomes (Table S1). This suggests that provisioning of tyrosine by symbionts, or tyrosine precursors, is of general importance across all bacteriocyte-associated symbioses of ants.

**Table 1:**
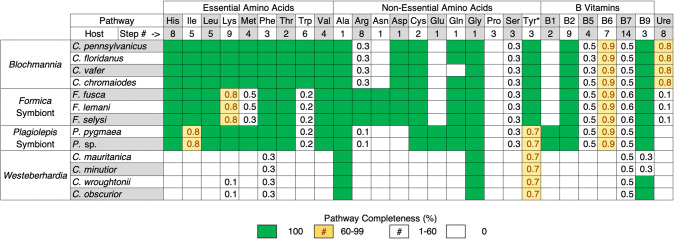
Comparison of the retention and losses of metabolic pathways for key nutrients across ant symbionts.

Pathways displayed are based on those that have been shown to play important roles in other ant and insect symbioses. Detailed breakdowns of these nutrient pathways along with analysis of other precursor, core metabolite synthesis, and transcriptional pathways, are available in Table S2. *Tyrosine is considered a non-essential amino acid because it can be synthesised by most eukaryotic hosts from phenylalanine.

The second major pattern emerging from our comparative analysis is that there are clear differences in the pathways lost or retained across symbionts (Tables 1 and S2). This is most evident when comparing *Blochmannia* with *Westeberhardia*, the latter of which has lost the capacity to synthesise most essential nutrients. The symbionts of *Formica* or *Plagiolepis*, in contrast, have retained the capacity to synthesise many of the same amino acids and B vitamins as *Blochmannia*, suggesting they may perform similar functions for their hosts. However, *Blochmannia* has retained more biosynthetic pathways, particularly those involved in the synthesis of essential amino acids. Previous experimental studies have confirmed that *Blochmannia* provisions hosts with essential amino acids [1]. The absence of several core essential amino acids in the *Formica* and *Plagiolepis* symbionts may reflect differences in the dietary ecology of the different ant genera. The retention of the full complement of essential amino acids biosynthetic pathways in the highly reduced genome of *Blochmannia* does however indicate it plays a more substantive nutrient-provisioning role for its hosts than the other ant symbionts we investigated.

Previous work on the extracellular gut symbionts of several arboreal ant lineages identified nitrogen recycling via the urease operon as a function that may be of key importance for ant symbioses [1, 2, 49, 50]. However, we do not find any evidence that the symbionts of *Formica, Plagiolepis*, or *Cardiocondyla* play a role in nitrogen recycling via the urease operon (Table 1). This suggests that nitrogen recycling may play an important role for more strictly herbivorous ants, such as *Cephalotes*. Our results indicate tyrosine supplementation by symbionts may be universally required for essential physiological process across a broader range of ant lineages.

### The origins and losses of symbioses in *Formica* and *Cardiocondyla*

We investigated the presence of the symbiont in phylogenetically diverse *Formica* and *Cardiocondyla* species to identify the evolutionary origins and losses of the symbiosis. Although the symbiont in *Plagiolepis* was present in *P. pygmaea* and two unknown *Plagiolepis* species we investigated, we did not have sufficient phylogenetic sampling to assess the origins of the symbiosis.

**Fig. 4:**
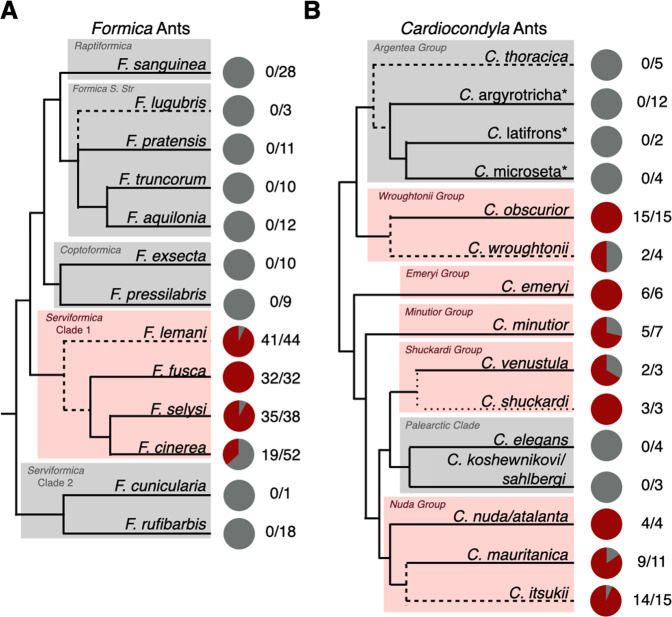
Phylogenetic distribution of symbionts in queens of *Formica* and *Cardiocondyla* ants. Pie charts represent the proportion of *Formica* (**A**) and *Cardiocondyla* (**B**) queens sampled that carried the symbiont (red) and those that did not (grey). Numbers represent the number of queens positive for the symbiont over the total number of queens sampled (intracolony infection frequencies in Table S5). See the supplementary material for the statistical testing of differences in prevalence within Serviformica Clade 1. The *Formica* phylogeny is based on [81] and the *Cardiocondyla* phylogeny is based on [83], with major clades highlighted. Dashed lines indicate species added to the original source phylogeny based on additional published phylogenies (specified in the Taxonomic Analysis section of the methods). Starred names are provisional names of a recognised morphospecies to be described by B. Seifert.

In *Formica*, we find the symbiont is restricted to a single clade in the paraphyletic Serviformica group (Fig. 4A). The species in this clade are socially polymorphic, forming both multi-queen and single-queen colonies [51]. Based on a previously dated phylogeny of *Formica* ants, we estimate the symbiosis originated approximately 12–22 million years ago [52]. In *Cardiocondyla*, the symbiosis is widespread throughout the genus. The prevalence of the symbiont in *Cardiocondyla*, in combination with its highly reduced genome, suggests it is a very old association that likely dates back to the origins of the ant genus some 50–75 million years ago [53]. The symbiont was also absent in two clades, the argentea and palearctic groups (Fig. 4B). This may represent true evolutionary losses in these clades. It may be that these losses are linked to a notable change in social structure in these two *Cardiocondyla* clades, having gone from the ancestral state of maintaining multi-queen colonies to single-queen colonies [54], however it is not clear how this could impact the symbiosis.

### Evidence of variation in colony-level dependence on symbionts

Observations from individual studies on *F. cinerea* and *F. lemani* [10, 11], as well as *Cardiocondyla obscurior* [8], reported rare cases of ant queens not harbouring their symbionts in nature. This called into question the degree to which these insects depend on symbionts for nutrients, and whether the symbiosis may be breaking down in certain host lineages. However, given the limited number of species and populations studied, it is unclear how often colonies are maintained with uninfected queens, and whether this varies across species, suggesting species may differ in their dependence on their symbiont. To answer this question, we assessed the presence of the symbionts in 838 samples from 147 colonies of phylogenetically diverse *Formica* and *Cardiocondyla* species collected across 8 countries.

Our investigation reveals the natural occurrence of uninfected queens is a widespread phenomenon in many *Formica* and *Cardiocondyla* species (Fig. 4). We confirmed the absence of symbionts in queens, and that they have not been replaced with another bacterial or fungal symbiont, using multiple approaches including diagnostic PCR, metagenomic and deep-coverage amplicon sequencing (Tables S3,  S4, Figs. S5, S6). *Wolbachia* was high in relative abundance, especially in *Formica* ants, but was not sufficiently present across samples to be a feasible replacement. There was also clear evidence of variation across host species. In *Formica*, queens and workers of *F. fusca* always carried the symbiont, whereas queens and workers of *F. lemani, F. cinerea*, and *F. selysi* showed varying degrees of individuals not carrying the symbionts (Fig. 4A, Table S5). A similar pattern can be seen in *Cardiocondyla*, where queens of several species, such as *C. obscurior*, always carry the symbiont, compared to lower incidences in other species (Fig. 4B). Klein et al [8] identified a single *C. obscurior* colony with uninfected queens in Japan. However, queens of this species nearly always carry the symbiont in nature.

The degradation and eventual loss of symbionts from bacteriocytes has been reported in males, and in sterile castes of aphids and ants [55], which do not transmit symbionts to offspring. In reproductive females, bacteriocytes may degrade as a female ages; however, symbionts are typically retained at high bacterial loads in the ovaries, as this is required to maintain the symbionts within the germline [31]. All of the symbiotic ant species we investigated maintain multi-queen colonies, and the vast majority had at least one queen, often more, within a colony that carried the symbiont (Table S5). We hypothesize that species that maintain colonies with uninfected queens may be able to retain sufficient colony-level fitness with only a fraction of queens harbouring the symbiont and receiving its nutritive benefits.

### Dependence on symbionts in a socioecological context

The retention of symbionts in queens and workers of some species, but not others, suggests species either differ in their dependence on symbiont-derived nutrients, or that symbionts have lost the capacity to make nutrients in certain host lineages. Our analysis of symbiont genomes did not reveal any structural differences, such as the disruption of metabolic pathways, which could explain differences in symbiont retention between host species (Table S2). This suggests differences in the retention of symbionts may reflect differences in host ecologies.

In ants, which occupy a wide range of feeding niches, reliance on symbiont-derived nutrients will largely depend on lineage-specific feeding ecologies. For example, several species of arboreal *Camponotus* ants have been shown to be predominantly herbivorous [56]. *Blochmannia*, in turn, has retained the capacity to synthesise key nutrients lacking in their plant-based diets, such as essential amino acids [1]. *Blochmannia* is also always present in queens and workers [31], which is a testament to the importance of these nutrients for the survival of its primarily herbivorous host [13]. In contrast, *Formica* and *Cardiocondyla* species are thought to have a more varied diet [14]. Diet flexibility and altered foraging efforts may therefore reduce their reliance on a limited number of symbiont-derived nutrients allowing colonies of some species to persist with uninfected queens in certain contexts. Silvanid beetles and grain weevils, for example, can survive in the absence of their tyrosine-provisioning symbionts [38, 57, 58] when provided nutritionally balanced diets, in the laboratory [57] or in cereal grain elevators [59, 60]. Similarly, studies on *Cardiocondyla* and *Camponotus* ants have shown they can maintain sufficient colony health in the absence of their symbionts, if provided a balanced diet [31, 61]. It would be interesting to know whether species of *Formica* and *Cardiocondyla* that always carry the symbiont in nature, such as *F. fusca* and *C. obscurior*, have more restricted diets with less access to nutrients such as tyrosine, as this may explain their dependence on their symbiont for nutrients and tendency to harbour them in queens.

Although it is unusual for bacteriocyte-associated symbionts to be absent in reproductive females, the fact that it is simultaneously occurring in phylogenetically diverse species from many locations suggests the symbiosis may have persisted in this manner over evolutionary time. Perhaps through diet flexibility colonies can be maintained with uninfected queens in some contexts, however we expect them to be disadvantaged in other ecological scenarios. Fluctuating environmental conditions may therefore eventually purge asymbiotic queens from lineages, allowing the symbiosis to be retained over longer periods of evolutionary time. The multiple-queen colony lifestyle in all symbiotic *Formica* and *Cardiocondyla* species we investigated may also provide an additional social buffer that limits the costs to individual queens being asymbiotic. Workers will still nourish larvae and queens without symbionts and colony fitness may be maintained through the reproductive output of nestmate queens that carry the symbiont. There may also be an adaptive explanation for the losses if, for example, metabolic costs to maintain the symbiosis trade off in a context dependent manner [44, 62, 63]. Under this scenario, maintaining a mix of infected and uninfected queens may benefit a colony by allowing for optimal reproduction under a broader range of environmental scenarios.

Our data suggest that symbiotic relationships can evolve to solve common problems but also rapidly break down if the symbiosis is no longer required, or potentially when costs are too high [44]. We have identified tyrosine provisioning as a possible unifying function across bacteriocyte-associated symbionts of ants. But we have also shown species can vary in how much they depend on symbionts for nutrients. Our results demonstrate that ants have a unique labile symbiotic system, allowing us to better understand the evolutionary forces that influence the persistence and breakdown of long-term endosymbiotic mutualisms.

### *Candidatus* Liliensternia hugann and *Candidatus* Jungenella plagiolepis

We propose the names *Candidatus* Liliensternia hugann for the *Sodalis*-allied symbiont found in *Formica*. The genus name is in honour of Margarete Lilienstern who first identified the symbiont [11]. The species name is derived from the combined first names of the first authors parents. Similarly, we propose the name of *Candidatus* Jungenella plagiolepis for the *Plagiolepis*-bound symbiont. The genus name is in honour of Hans Jungen who originally discovered the symbiont [10], and the species name is derived from *Plagiolepis*, the genus in which the symbiont can be found.

## Materials and methods

Detailed protocols for each of the following sections are available in the Supplementary Materials, under Supplementary Methods.

### Metagenomic sequencing and analysis

Single queens from 16 different species of *Formica*, *Plagiolepis*, and *Cardiocondyla*, were sequenced using the HiSeq 4000 (Illumina). Of these samples, 8 queens from 3 *Formica* species (*fusca*, *lemani*, *selysi*), 2 *Plagiolepis* species (*pygmaea*, spp.), and 3 *Cardiocondyla* species (*minutior*, *mauritanica*, *wroughtonii*) carried the symbiont of interest at sufficient coverage for metagenomic assembly. Raw reads were trimmed and then subjected to two rounds of filtering, metagenomic binning, and assembly using SPAdes V3.11.1 [64]. Blobplots of contigs graphed by coverage and GC content and coloured by taxonomic identification are available as Fig. S7. Genomes were annotated using Prokka V1.14.6 [65]. Pathway completeness was assessed using manual curation and the metacyc resources for *E. coli* str. K-12 [66]. Single copy orthologs were identified using Orthofinder V2.2.7 [67]. Enriched functional categories and pathways were identified using David [68, 69].

### Taxonomic analysis and congruence testing

The phylogeny of ant genera (Fig. 1) is based on a previous analysis [70] with additional tip placements based on [53]. The phylogeny of endosymbiont species (Fig. 2) is a pruned version of the full phylogeny (Fig. S8). The full phylogeny was created using the protocol developed by Husnik et al. [71] to optimally resolve phylogenetic relationships of gammaproteobacteria endosymbionts, because they are known to be difficult to resolve. We used GtoTree [72] to generate an alignment of 72 core gammaproteobacterial genes. This alignment was then recoded using Dayhoff6 recoding using phylogears2 v2.0.x [73]. The phylogeny was then generated with phylobayes v4.1b [74–77] using cat and gtr settings. Model fit and convergence was assessed using Tracer v.1.2.7 [78] and Phylobayes’s bcomp function. We followed Phylobayes’s recommendations that maxdiff should be less than 0.3 (maxdiff was 0.255185). A consensus tree was generated using two chains that had run for 30,000 iterations with a burn in of 3000, sampling every 10 iterations. Free-living relatives of symbionts were selected based on a previous phylogeny of the *Enterobacteriales* [71, 79].We tested congruence between host and symbiont phylogenies using TreeMap 3b [80].

The phylogeny of ant species (Fig. 4) is based on a previous analysis of ant species [81] with additional tip placements based on a phylogeny of cytochrome B sequences of ants [82] (including sequences from individuals we had sequenced for *Formica* (Fig. S10)) and a phylogeny for *Cardiocondyla* [83] with additional tip placements based on a different phylogeny of *Cardiocondyla* [84].

### FISH microscopy

FISH was performed on eggs, queen guts, queen ovaries (whole mount), and on larvae (cytological sections), using 16 S rRNA oligonucleotide probes specifically targeting the symbionts. The probes used were 5’-Cy3-CGCTACACCTGAAATTCT-3’ for the *Formica* symbiont, or 5’-Cy3-CGCTACACCTGGAATTCT-3’ for the *Plagiolepis* symbiont. Following overnight incubation at room temperature, the samples were mounted using Vectashield hardset antifade mounting media with DAPI and visualised using a Leica DMRA2 epi-fluorescent microscope. Detailed protocol can be found in the Supplementary Materials, under Supplementary Methods.

### Symbiont screening procedure

We screened 838 individuals, a mixture of queens and workers, from 147 colonies across 29 species for the presence of symbionts using a combination of diagnostic PCR screening and 16 S rRNA deep coverage sequencing (Table S3). Diagnostic PCRs were carried out by amplifying the symbiont 16 S rRNA genes from total genomic DNA extracted from individual ants. Custom primer pairs (Table S5) were used for screening *Sodalis* and *Westeberhardia*, respectively. Positive queen diagnostic PCR results were confirmed using Sanger sequencing.

For 16 S rRNA deep coverage sequencing, the 515 F/806 R primer pair [85] was used to amplify the V4 region of the 16 S rRNA gene in two runs of 16 S rRNA sequencing in 177 *Cardiocondyla* and *Formica* samples (Table S3). Additionally we conducted a run of ITS fungal sequencing using the ITS5/5.8S fungi primer pair [86] (Table S3), to investigate whether any fungal symbiont replacement could be detected. Samples contain trace amounts of symbiont reads after multiplex 16 S rRNA sequencing were reanalysed using targeted qPCR, and diagnostic PCR, with symbiont-specific primers, to confirm the symbiont was indeed absent. The qPCR validation was conducted on samples from symbiont carrying, and non-symbiont carrying species.

16 S rRNA gene sequencing data were analysed using Mothur v.1.41.3 [87], to cluster reads into OTUs at 99% similarity. ITS sequencing data were analysed using USEARCH [88] and UPARSE [89] to cluster reads in zero-radius OTUs (ZOTUs). Data were then processed using R to remove OTUs/ZOTUs at below 1 percent relative abundance in a sample and generate visualizations.

We chose a threshold of 1 percent relative abundance, as we found the symbiont was not detectable by targeted PCR or qPCR using symbiont-specific primers at this level.

## Supplementary information

Supplementary Materials

Supplementary Tables

## Data Availability

All data collected in association with this paper, alongside associated genome assemblies, are available under BioProject accession PRJNA639935.
