# Supplementary material for: Convergent evolution of a labile nutritional symbiosis in ants
Source: ISME J. 2022 Jun 14;16(9):2114–22. doi: 10.1038/s41396-022-01256-1 (PMC9381600; doi:10.1038/s41396-022-01256-1)
Supplement: Supplementary file 1 — Supplementary Materials [file 41396_2022_1256_MOESM1_ESM.docx]

**Supplementary Information for**

Convergent evolution of a labile nutritional symbiosis in ants

Raphaella Jackson, David Monnin, Patapios A. Patapiou, Gemma Golding, Heikki Helanterä, Jan Oettler, Jürgen Heinze, Yannick Wurm, Chloe K. Economou, Michel Chapuisat, Lee M. Henry

Lee Henry

Email: l.henry@qmul.ac.uk

**This file includes:**

Supplementary text: Supplementary Methods

Supplementary text: Supplementary Results

Figures S1 to S9

Legends for Datasets S1 to S10

SI References

**Other supplementary materials for this manuscript include the following:**

Datasets S1 to S10

**Supplementary Methods**

**DNA Extraction**

Genomic DNA was extracted from whole ants using the Qiagen DNeasy Blood and Tissue Kit (Qiagen, Venlo, Netherlands) according to the manufacturers’ protocol or using a phenol chloroform extraction method. Sample extraction method is listed in Table S3.

**Metagenomic Sequencing**

We performed metagenomic sequencing on ants, and the symbionts they harbour, by sequencing individual queens belonging to the genera *Cardiocondyla*, *Plagiolepis,* and *Formica*. We sequenced the genomes of nine *Cardiocondyla* species (*C. minutior, C. mauritanica, C. obscurior* (two samples: one from Tenerife and the other from Japan)*, C. “argyrotricha”, C. elegans, C. venustula, C. wroughtonii, C. nuda/atalanta, C. thoracica*), four *Formica* species (*F. fusca*, *F. lemani*, *F. selysi, F. cinerea*), and two species of *Plagiolepis* ants (*P. pygmaea*, and an unknown species of *Plagiolepis*, hereafter referred to as *Plagiolepis* sp.).

Genomes were sequenced in two runs. The first run consisted of all the *Cardiocondyla* samples excluding *C. minutior* and *C. mauritanica,* which were included with the *Formica* and *Plagiolepis* samples in the second run. The first, all *Cardiocondyla* samples run, used the Nextera XT DNA library kit, the second run used the NEBNext Ultra II DNA kit. Both runs were sequenced by multiplexing samples on a single lane of the Illumina Hiseq 4000 machine using 150bp paired-end reads.

**Metagenomic Assembly, Gene and Functional Annotation**

Paired-end reads were trimmed to remove adaptors and subjected to basic quality control, using a sliding window length of 4 with a minimum quality of 20 and dropping reads under 36 basepairs in length, using Trimmomatic V0.38 (1). We then assembled reads using SPAdes V3.11.1 (2) with default parameters in assembler only mode. The original reads were mapped back to resulting contigs using bwa mem V0.7 (3).

We used Blobtools V1.1.1 (4)(10.5281/zenodo.845347) for metagenomic binning based on coverage and GC content, to assess the microbial communities present in the samples and identify and bin symbionts of interest. Both DIAMOND v0.9.24.125 (5), with the NCBI nr database, and Blastn (6), with the NCBI nt database, were used to assign taxonomic identifications to the contigs from the first round of assembly. Based on these results, we found clear evidence of the symbionts in all the *Plagiolepis* samples, all the *Formica* samples except from *F. cinerea*, and only the samples from *C.* *mauritanica, C. minutior, the Japanese* sample of *C. obscurior*, and *C.* *wroughtonii* (*Sodalis*-like symbionts for *Formica* and *Plagiolepis* samples, *Westeberhardia* for *Cardiocondyla* samples). The symbiont carrying sample of *C. obscurior* from Japan did not carry the symbiont of interest at sufficient coverage (greater then 2X coverage). to attempt a proper assembly. It was unclear if the symbiont was present in the *C. obscurior* sample from Tenerife, as only two approximately 500 base pair contigs were identified as *Westeberhardia* and there was no evidence of the symbiont other than this. Both *C. obscurior* samples were from long-standing lab colonies (10+ years) where symbiont losses occasionally occur.

In our non-symbiont carrying *Cardiocondyla* queens, some of which were from species that normally carry the symbiont (e.g. *C. nuda/atalanta*, *C. venustula*), no contigs were identified as belonging to *Westeberhardia*. Additionally, all samples except *C. nuda/atalanta* had no more than 10,000 base pairs attributed to the order *Enterobacterales*. *C. nuda/atalanta* had a large number of base pairs attributed to the order *Enterobacterales* but this was largely the result of having a strain of *Arsenophonus*. In our non-symbiont carrying *F. cinerea* sample there were no contigs identified as *Sodalis* or *Sodalis*-like species. Additionally, we only found two contigs (1,173 total base pairs) attributed to *Enterobacterales* (they were ID’d as *Salmonella* and *Klebsiella*). Finally we used Kraken2 to classify reads from all samples in order to check for symbiont presences. We used the full Kraken2 bacterial, fungal, and common contaminants databases alongside all available ant genome assemblies and the existing *Westeberhardia* assembly for a classification database. We considered all reads classified as *Sodalis* species to be potential symbiont reads in *Formica* and *Plagiolepis* species. We considered all reads classified as *Candidatus* Westeberhardia to be potential symbiont reads in *Cardiocondyla* species. Due to a high level of divergence in *Westeberhardia* strains/species levels of reads identified as *Westeberhardia* varied by host species distant from *C. obscurior* but there was still a clear difference between symbiotic and asymbiotic individuals.

In order to improve the assembly of the symbiont of interest, samples carrying the symbiont at sufficient levels of coverage were put through a second round of assembly. The contigs from the first assembly were classified using DIAMOND v0.9.24.125 to conduct a blastx search against the NCBI’s nr database. This search was taxonomy restricted to taxid 543 (Family:Enterobacteriaceae). All reads mapping to contigs that reported hits in this search were retrieved using samtools V1.9 (7) and then reassembled using SPAdes, this time in careful mode with kmer sizes of 33,55,77,99, and 127. The resulting contigs were then classified using both blobtools metagenomic binning and DIAMOND with a taxonomy restricted search to 84565 (Genus:Sodalis) for the *Formica* and *Plagiolepis* samples and restricted to 543 (Family: Enterobacteriaceae) for the *Cardiocondyla* samples. All contigs then manually inspected to compare metagenomic binning and DIAMOND search results to determine whether they belonged to the symbiont of interest. Blobplots of contigs graphed by coverage and GC content as well coloured by taxonomic identification are available as figure S7.

Each genome was annotated using Prokka V1.14.6 (8) under default parameters, and pseudogenes were identified using a combination of DFAST V1.2.3 (9), using the options for prodigal (10) and blastp, using the gene annotations of *Escherichia coli* str. K-q12 substr. MG1655 (11) as a reference, and PGAP (12) under default settings.

Pathway completeness was assessed using manual curation and the metacyc resources for *E. coli* str. K-12 (13). Amino-acid and B vitamin synthetic pathways, alongside the completeness of the urease operon were selected because they have been shown to play key roles in other ant and insect symbiosis. Additional pathways were selected on the basis that they either play essential roles for bacterial survival or contribute precursors to the synthesis of the amino acids and vitamins (Table S2). Quorum sensing and type III secretion system genes were also included as they have been found to play key roles in pathogenicity and independent transmission of closely related *Sodalis* bacteria (14,15).

**Intergenomic Comparisons**

To compare genome structure between the *Sodalis*-like endosymbionts in *Formica* and *Plagiolepis* ants, we identified single copy orthologs between the assembled genomes using Orthofinder V2.2.7 (16) and then used a customised version of a script created by Filip Husnik for the tool Processing (available from <https://github.com/filip-husnik/genome-plots-processing>) as originally used in (17) to generate the visualisation. Processing is a programming language which allows the user to create custom visualizations (https://processing.org).

To compare gene sets among symbiont lineages we used Orthofinder V2.2.7 to group the genes by orthologous and identify which orthologs were present in each genome. We then used the programme David’s (18,19) functional annotation clustering under the option for high classification stringency to identify enriched terms and pathways in the list of genes. We used *E. coli* strain K-12 as a background. A total of 284 genes were input into David out of which 117 were successfully clustered into 16 groups based on similarity of functional annotation. The functional terms associated with each group were inspected for terms associated with nutrient provisioning by symbionts.

**Taxonomic Analysis**

The phylogeny of *Westeberhardia* symbionts used in the co-phylogeny in Figure S2 is based on three genes (16S rRNA, cvrA and groL). Sequences were obtained from *Cardiocondyla* ants (*C. emeryi, C. itsukii, C. mauritanica, C. minutior, C. nuda/C. atalanta, C. obscurior, C. shuckardi, C. venustula and C. wroughtonii*), either from the genomes sequenced in the present study or through PCR and Sanger sequencing using the following primers: 16S F2, 16S 188 F3, 16S R5, 16S 980 R1, CvrA92F, CvrA 494F, CvrA 1013R, CvrA 1196R, groL233F, groL 257F, groL 1216R and groL 1473R (Primers sequences listed in Table S6). A touchdown PCR program was used, with amplification conditions as follows: initial denaturation of 94 °C for 2 min, followed by 11 cycles of 94 °C for 20 s, 56 °C (dropping by 1 °C each cycle) for 50 s, and 72 °C for 30 s, followed by 25 cycles of 94 °C for 2 min, 45 °C for 50 s, and 72 °C for 2 min and a final extension of 5 min at 72 °C. The alignment was performed using MUSCLE (20) and tree was built using PhyML(21) using default parameters, substitution model HKY85, with a bootstrap value of 100.

**FISH Microscopy**

We performed whole mount FISH and FISH on cytological sections using ants sampled at different life stages from laboratory colonies. Laboratory colonies were purchased through [www.antsrus.com](http://www.antsrus.com) (*F. fusca* and *Plagiolepis* sp.), or collected from field sites around Helsinki, Finland, (*F. fusca*, *F. cinerea*).

Whole mount FISH was performed on eggs and adult queens of *F. fusca* and *Plagiolepis* sp. following a protocol adapted from Koga (22) and Sanders (23). The gut and ovaries of the queen were dissected in PBS (Phosphate Buffer Saline). Eggs, dissected ovaries and dissected gut were fixed for 2 hours in a formaldehyde solution (4% in PBS). The samples were then washed in PBS and then in 75% ethanol, bleached in an alcoholic H_2_O_2_ solution (80% ethanol, 14% H_2_O, 6% H_2_O_2_) for 3 days, changing the solution each day, and washed again in 90 % ethanol. The samples were then washed 4 times (30 minutes each) in PBSTx (PBS with 0.3% of Triton X-100), and then 3 times (5 minutes each) in pre-hybridisation buffer (20 mM Tris-HCl at pH 8.0, 0.9 M NaCl, 0.01% sodium dodecyl sulfate and 30% formamide). The samples were then incubated overnight at room temperature in hybridisation buffer, i.e. prehybridisation buffer supplemented with 100 mM of a specific fluorescent probe (ordered from Eurogentec, Seraing, Belgium), either 5'-Cy3-CGCTACACCTGAAATTCT-3' for the *Formica* symbiont, or 5'-Cy3-CGCTACACCTGGAATTCT-3' for the *Plagiolepis* symbiont. Following washes in PBSTx, the samples were mounted on slides in Vectashield hardset antifade mounting media with DAPI. Mounted samples were visualised using a Leica DMRA2 epi-fluorescent microscope. Monochrome pictures were obtained using a Hamamatsu Orca camera and the Volocity 6.3.1 software, and final colour images were obtained using ImageJ.

For FISH on cytological sections on larvae, tissues were fixed, bleached and washed as previously described. They were then embedded in paraffin. Prior to the embedding, they were washed in absolute ethanol (4×10 min) xylenes (2×2 min), and paraffin (3×1 hour). Sections (9 µm) were obtained using a microtome Leica RM2145 and placed on polysine slides. The slides were dewaxed in xylenes (2×5 min), washed in absolute ethanol, 96% ethanol and 70% ethanol (5 min each) and in PBS (2×5 min). The sections were then covered with prehybridisation buffer for one hour, and then with hybridisation buffer overnight. Mounting and imaging were performed as described above.

**Diagnostic PCR and Sanger Sequencing**

All PCR reactions for sanger sequencing were performed using MangoMix (Meridian Bioscience, Cincinnati, US). Diagnostic PCRs were carried out by amplifying the symbiont 16S rRNA genes from ant genomic DNA. Custom primer pairs for Sodalis 16S diagnostic F2/Sodalis 16S R2 and WeBh2_F/WeBh2_R, were used for screening *Sodalis* and *Westeberhardia*, respectively (Table S6). The following cycling conditions were used for *Sodalis* diagnostic PCRs: initial denaturation at 94 °C for 2 min, then 30 cycles of 94 °C for 1 min, 53 °C for 1 min, 72 °C for 1 min, and a final extension of 72 °C for 5 min. The following cycling conditions were used for *Westeberhardia* diagnostic PCRs: initial denaturation at 94 °C for 2 min, then 35 cycles of 94 °C for 1 min, 54 °C for 1 min, 72 °C for 1 min, and a final extension of 72 °C for 5 min. Ant species were confirmed by amplifying the cytochrome c oxidase I (COI) mitochondrial gene using the forward primer, Jerry, with reverse primer Ben degenerate (24,25). A touchdown PCR program was used, with amplification conditions as follows: initial denaturation of 94 °C for 2 min, followed by 11 cycles of 94 °C for 20 s, 56 °C (dropping by 1 °C each cycle) for 50 s, and 72 °C for 30 s, followed by 25 cycles of 94 °C for 2 min, 45 °C for 50 s, and 72 °C for 2 min and a final extension of 5 min at 72 °C.

**16S rRNA and ITS Sequencing**

Two separate runs of 16S rRNA sequencing were conducted. See Table S3 for samples identified by run.

In run 1, we used the 515F/806R primer pair (26) to amplify the V4 region of the 16S rRNA gene (Table S6). All PCR reactions were performed using Q5 High-Fidelity master mix (New England Biolabs, Ipswich, Massachusetts, USA). For the first stage PCR, amplification conditions were as follows: initial denaturation at 98°C for 30 s followed by 25 cycles of 98 °C for 10 s, 50 °C for 15 s, 72 °C for 20 s and a final extension of 72 °C for 5 min. PCR clean-ups were performed using AMPureXP beads (Beckman Coulter Life Sciences, Indianapolis, United States) and then a second stage PCR was carried out to attach dual indices and illumina sequencing adapters. Second stage PCR conditions were as follows: 95 °C for 3 min followed by 8 cycles of 98 °C for 20 s, 55 °C for 15 s, 72 °C for 15 s and a final extension of 72 °C for 5 min. A second PCR clean-up using AMPure XP beads was performed to clean up the libraries before quantification. Individual PCR products were quantified using the Qubit dsDNA HS Assay Kit (Thermo Fisher Scientific, Massachusetts, United States) and the libraries were then normalized and pooled. The pool was sequenced at Edinburgh Genomics (University of Edinburgh) on an Illumina MiSeq (paired-end, 2 x 250 bp reads).

For run 2, the 515F/806R primer pair (26) was used to amplify the V4 region of the 16S rRNA gene. The ITS5/5.8S_fungi primer pair (27) was used for the amplification of fungi (Table S6). All PCR reactions were performed using Q5 High-Fidelity master mix. PCR reactions were carried out using amplification conditions as follows: initial denaturation at 98 °C for 30 s followed by 25 - 35 cycles of 98 °C for 10 s, 50 °C (16S rRNA primers) or 62 °C (fungal primers) for 15 s, 72 °C for 20 s and a final extension of 72 °C for 5 min. PCR products were submitted to the Centre for Genomic Research (University of Liverpool) for addition of indices and adapters, and pooling of libraries. Sequencing was then carried out on a MiSeq (paired-end, 2 x 250 bp reads).

**16S rRNA/ITS Analysis Method**

Adaptor sequences were removed using Trimmomatic V.0.38 (1) using default parameters for adaptor removal, ILLUMINACLIP:(NexteraPE-PE.fa/TruSeq3-PE-2.fa):2:30:10:4:true.

For the 16S rRNA analysis, Mothur v.1.41.3 (28) was used to cluster the sequences into OTUS at a 99 percent level of similarity. The procedure used was a customized version of the MiSeq SOP available from the Mothur wiki page. The data, which was not rarefied or subsampled, was then transferred to R for further analysis. In R, all OTUs that accounted for less than 1 percent of total OTU content in an individual sample were filtered out of that sample. The OTU of the *Sodalis*-like species was determined using Blast and aligning to the 16S rRNA regions of the *Sodalis*-like genomes. Graphs were then generated using ggplot2 (29).

For the ITS analysis, USEARCH (30) was to merge, filter, and process reads before using UPARSE (31) to cluster the reads into zero-radius OTUs (ZOTUs). USEARCH was then used to assign taxonomy to these ZOTUs using the v2 of the RDP ITS database (32).

**Symbiont losses within Serviformica Clade 1**

In R, we tested for significant differences in symbiont prevalence within Serviformica Clade 1 using a binomial glm and the glht function (multcomp package) to perform Tukey post hoc tests.

**Supplementary Results**

**Symbiont losses within Serviformica Clade 1**

There were significant differences in symbiont prevalence within Serviformica Clade 1 (χ²_3_=10.967, p=0.01191). The prevalence in *F. cinerea* was significantly lower than in *F. fusca* (z=2.747, p=0.0301), *F. lemani* (z=-2.714, p=0.0334) and *F. selysi* (-2.597, p=0.0461). No other significant difference was found.

**
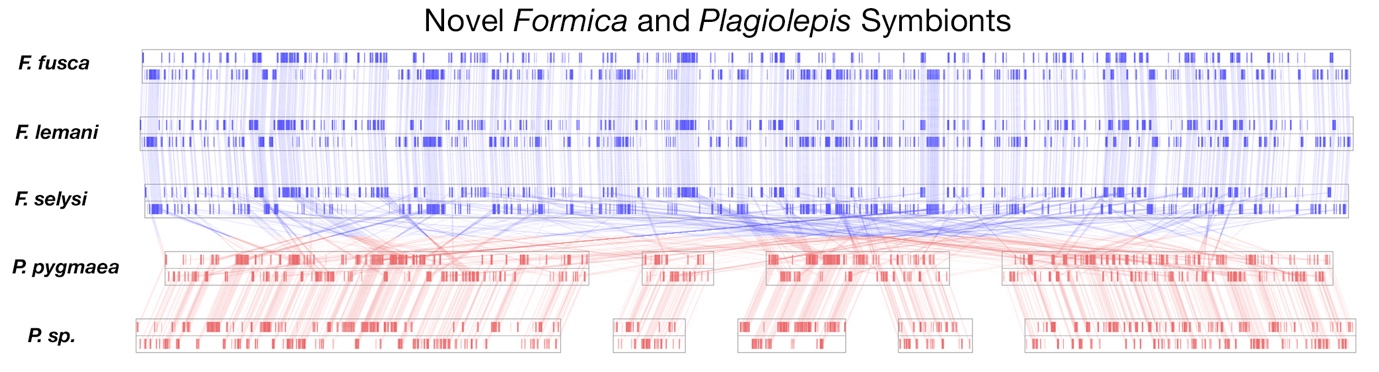
**

**Figure S1:** Comparing the order of single copy orthologs between the *Sodalis*-like symbionts in *Formica* and *Plagiolepis* ants. Gene order is conserved in symbiont lineages from ant species within the same genus, but not between ant species of different genera.

**
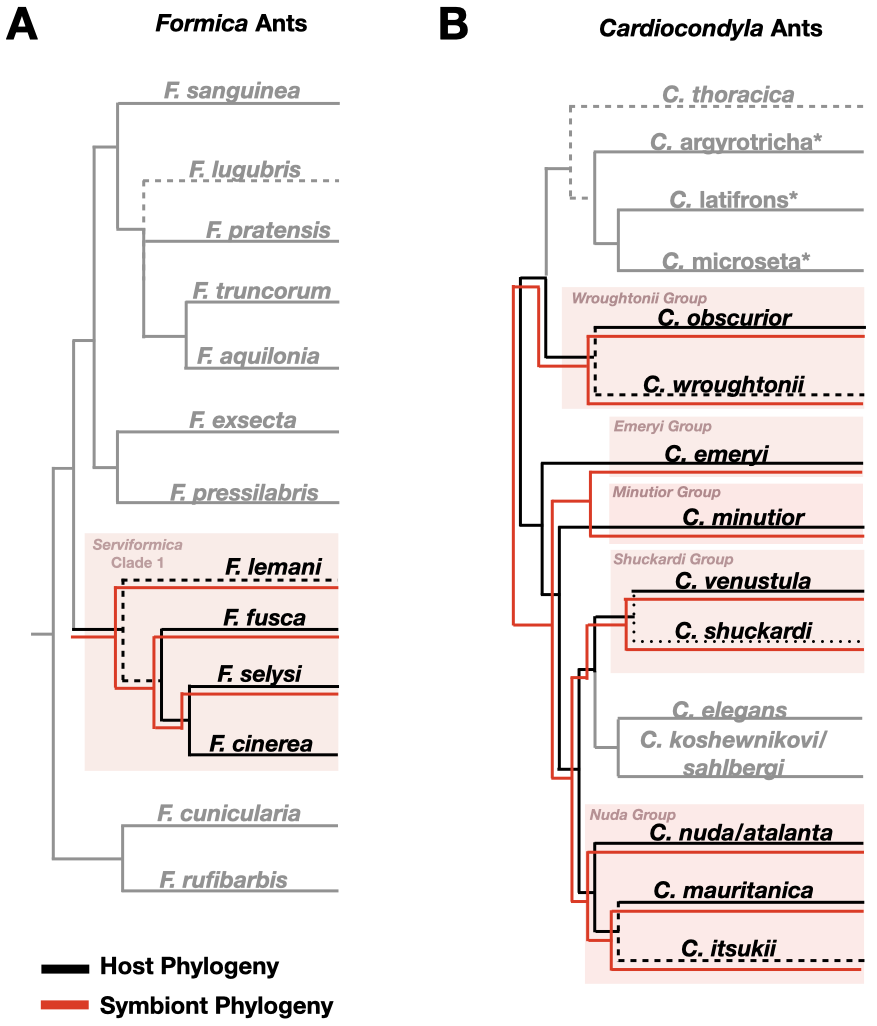
Figure S2:**  Co-phylogenies of *Formica* and *Cardiocondyla* ant species and their symbionts. Host phylogeny is indicated in black and is identical to that found in Figure 1. Symbiont phylogeny is indicated in red. The symbiont phylogeny for the *Sodalis* symbiont in *Formica* species is identical to that found in Figure 2. The symbiont phylogeny for *Westeberhardia* in *Cardiocondyla* is based on 3 genes: 16S rRNA, cvrA and groL, detailed procedure for this phylogeny is found in supplementary methods section: Taxonomic Analysis.

**
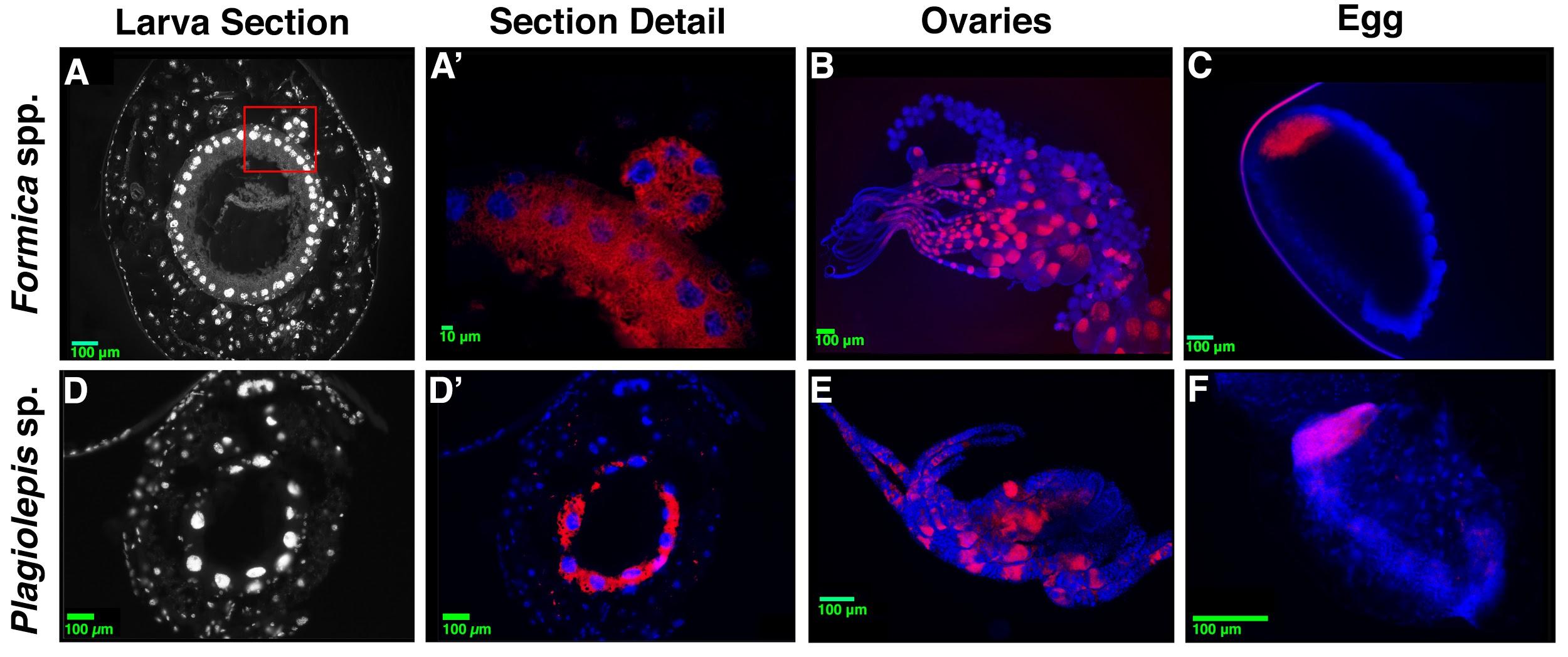
Figure S3:**  Fluorescent *in situ* hybridisation generated images comparing the localization of *Sodalis*-like symbionts in different regions of *Formica* and *Plagiolepis* ants. A-A’. FISH on transversal cytological sections of *Formica cinerea* larva. DAPI staining only (A), DAPI staining in blue, symbiont stained in red showing localisation of symbiont in bacteriocytes and a bacteriome (A’). B-C. Whole mount FISH of *Formica fusca*: queen ovaries (B) and egg (C). DAPI staining in blue, symbiont stained in red. D-D’. FISH on transversal cytological sections of *Plagiolepis* sp. larva. DAPI staining only (D), DAPI staining in blue, symbiont stained in red (D’), demonstrating symbionts are localised in cells surrounding the midgut. E-F. Whole mount FISH of *Plagiolepis* sp.: queen ovaries (E) and egg (F). DAPI staining in blue, symbiont stained in red, showing the symbiont is localised in a similar manner to that which is found in *Formica* ants.

**
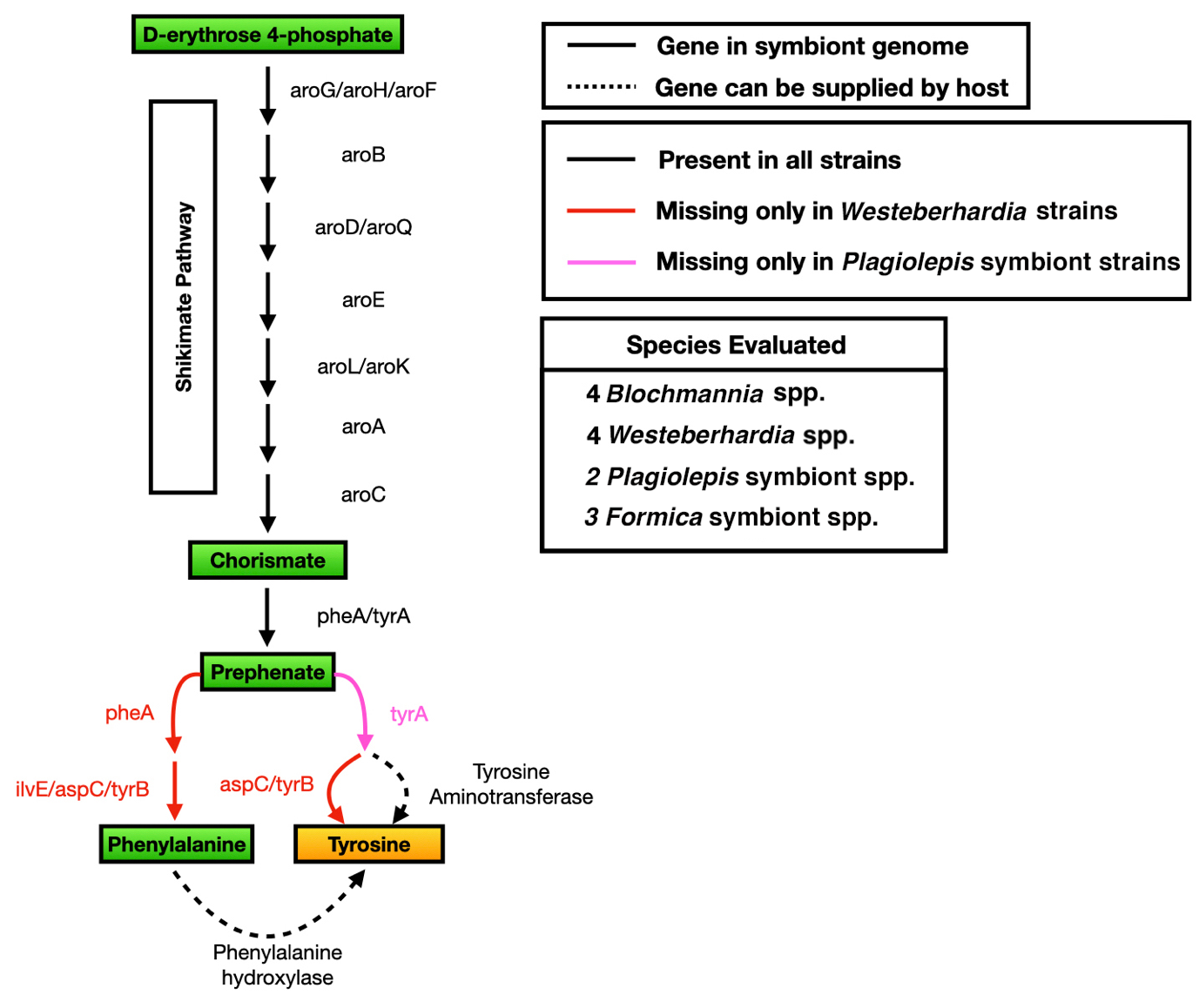
**

**Figure S4:** Schematic diagram comparing pathways for synthesising tyrosine and phenylalanine across bacteriocyte-associated symbionts of ants.

**
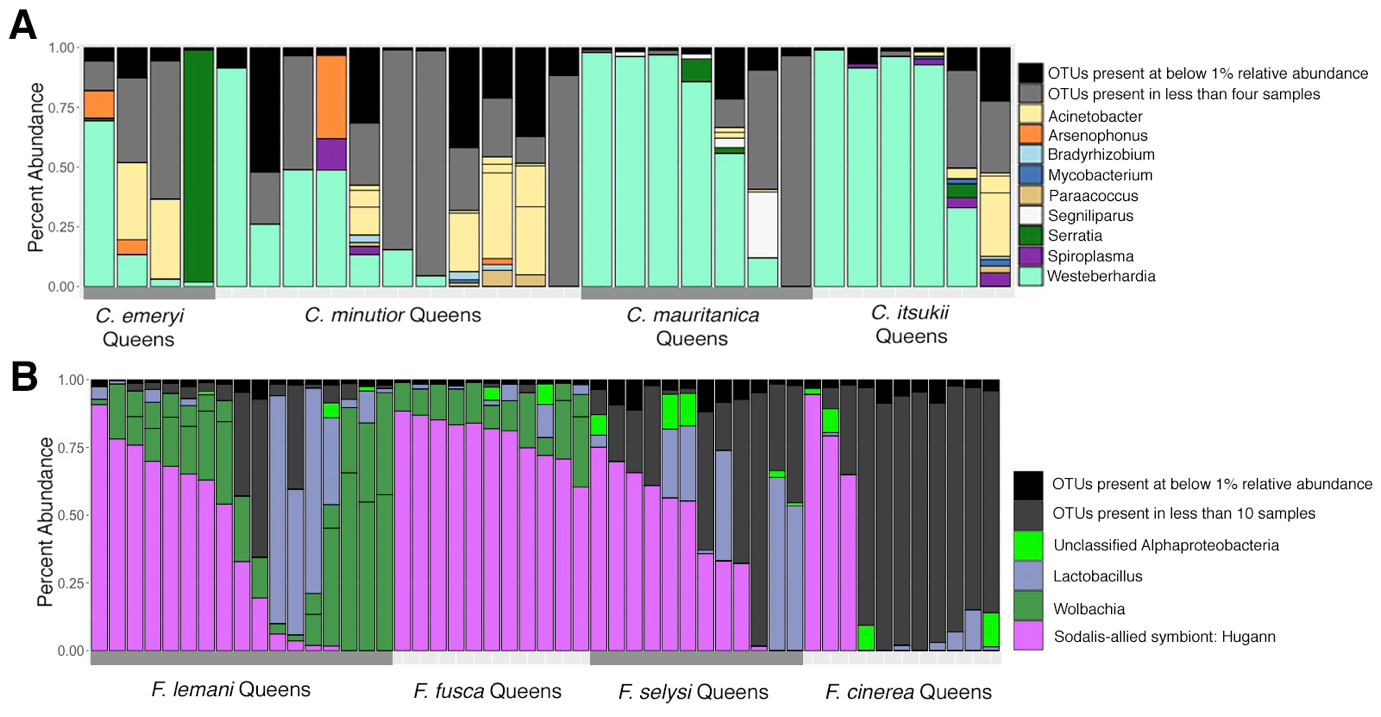
**

**Figure S5:** 16S rRNA relative abundance of bacterial OTUs in queens of *Cardiocondyla* (A) and *Formica* (B) species. To aid in interpretation of the data OTUs present at less than one percent relative abundance are grouped together and coloured black. Additionally, OTUs present in less than 10 samples in *Formica* species or less than four samples in *Cardiocondyla* species are grouped together and coloured grey. 10 samples and 4 samples were chosen as one less than the number of samples in the most lowly sampled species group in *Formica* and *Cardiocondyla* respectively. The data used to create these visualizations is available in Tables S7-S11.

**
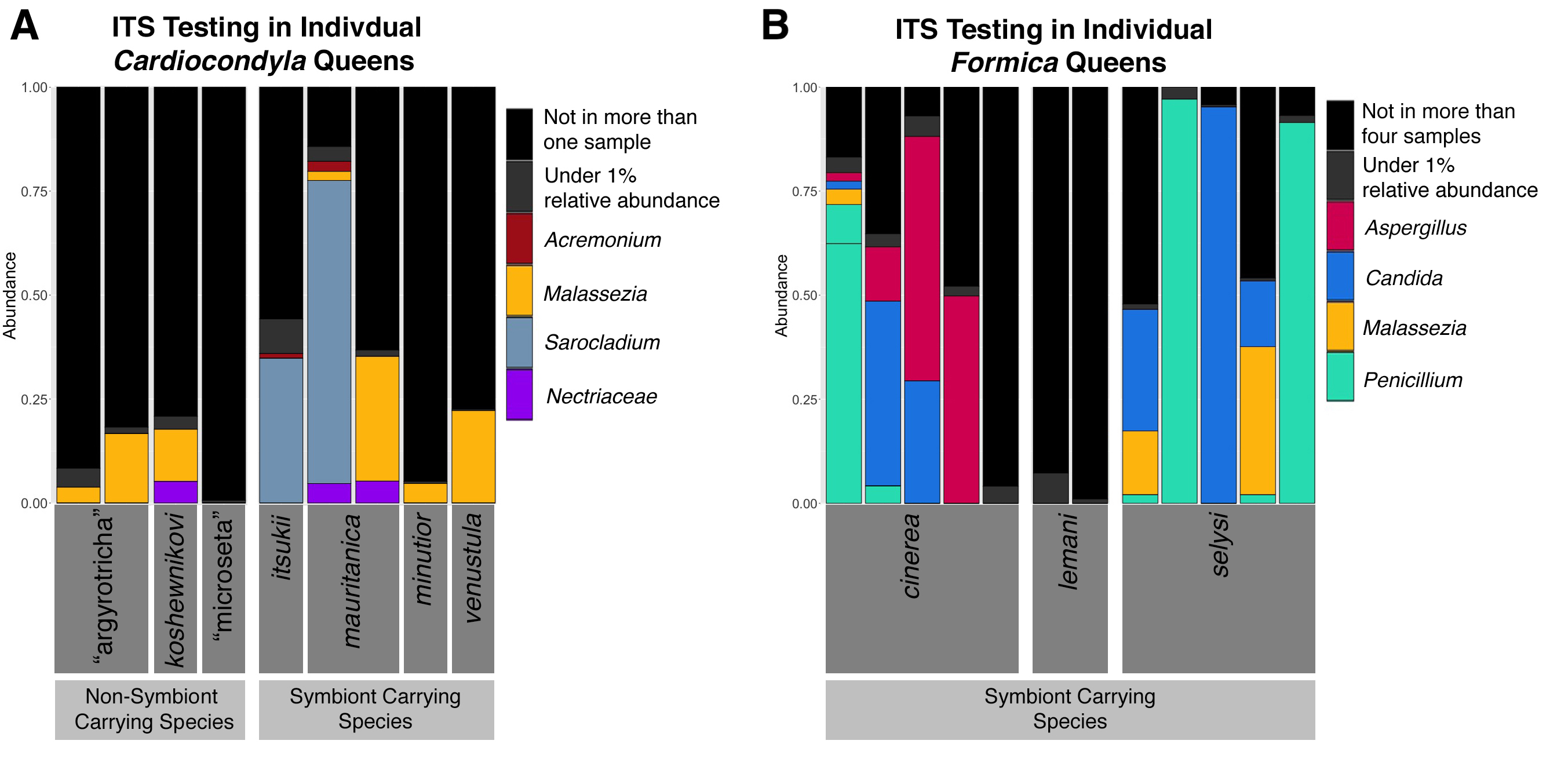
**

**Figure S6:** ITS Relative Abundance of Fungal ZOTUs in *Cardiocondyla* (A) and *Formica* (B) Queens that were found to not carry the *Sodalis*-like or *Westeberhardia* symbionts to assess possible fungal replacements (several individuals with the symbiont were included for comparison). The absence of the *Sodalis*-like symbiont or *Westeberhardia* was initially confirmed using a combination of diagnostic PCR and 16S rRNA sequencing data in samples found in Table S3. For legibility, a cut-off is used in each of visualizations to exclude ZOTUs that were not consistently present in all queens found without the symbiont, suggesting an evolutionary replacement. In (A), ZOTUs must be present in more than one sample from a symbiont carrying species to be visualized. In (B), ZOTUs must be present in at least 4 samples within a species to be visualized. C. “argyrotricha” and C. “microseta” are provisional names of recognized morphospecies to be described by B. Seifert.


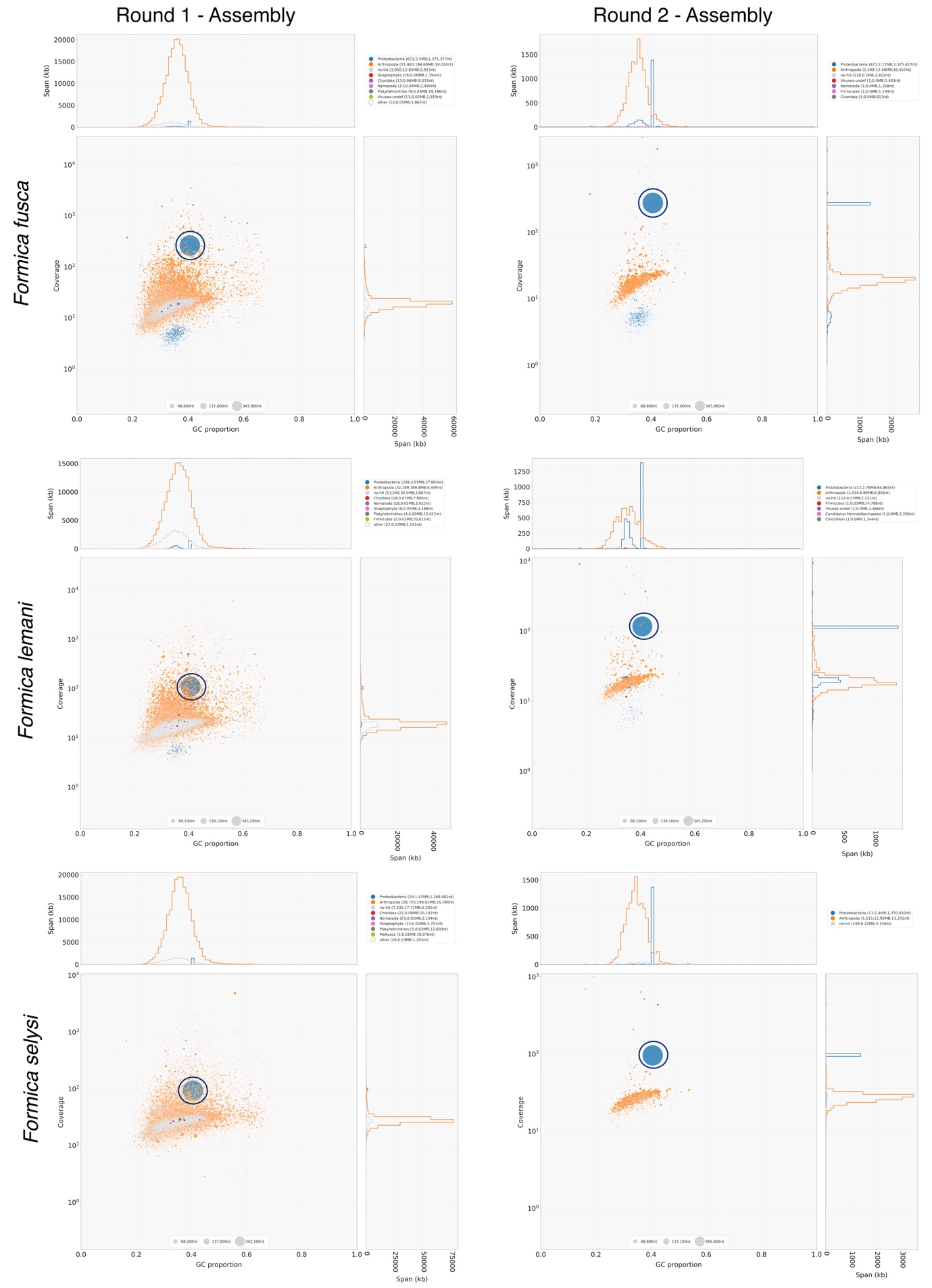


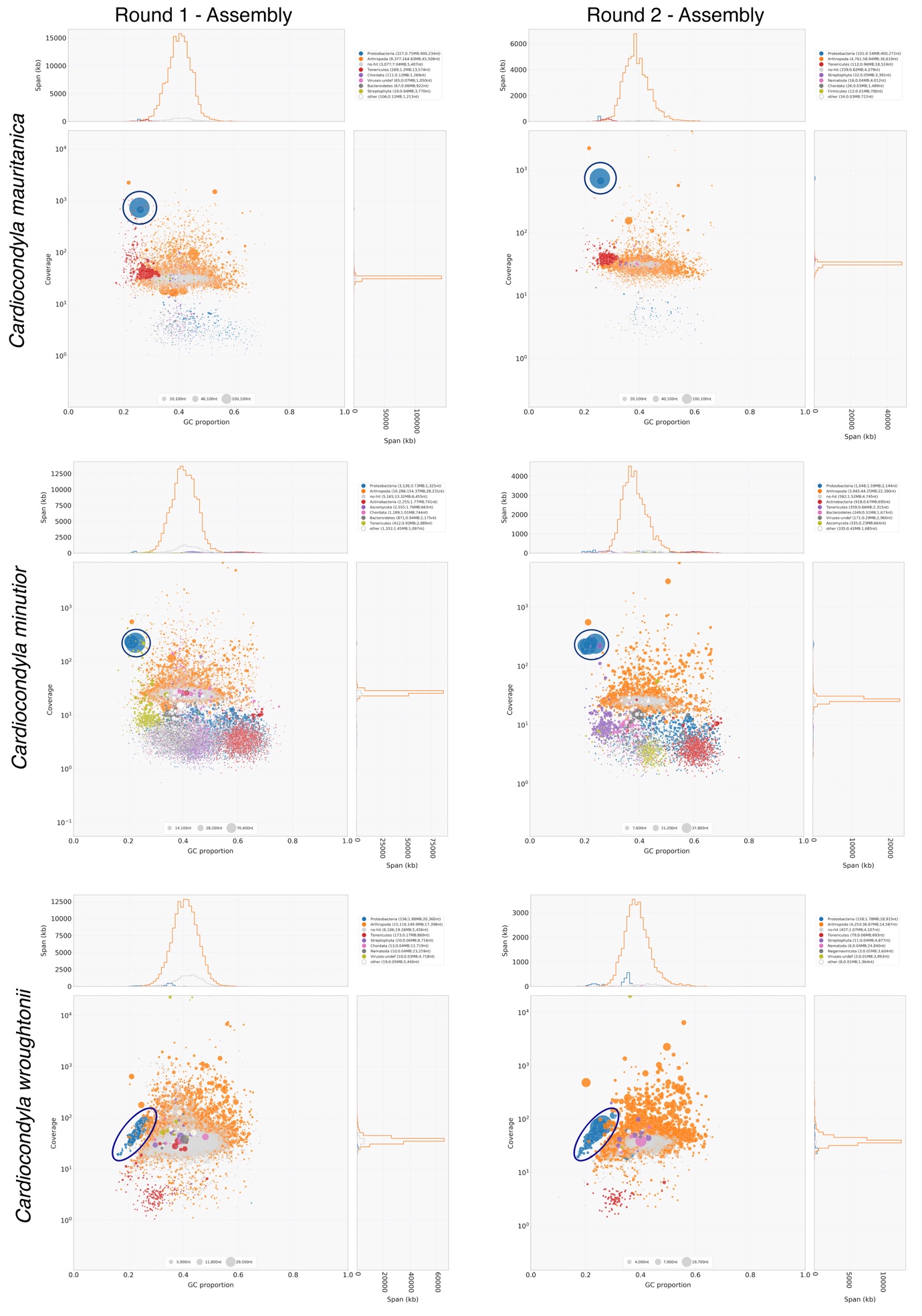

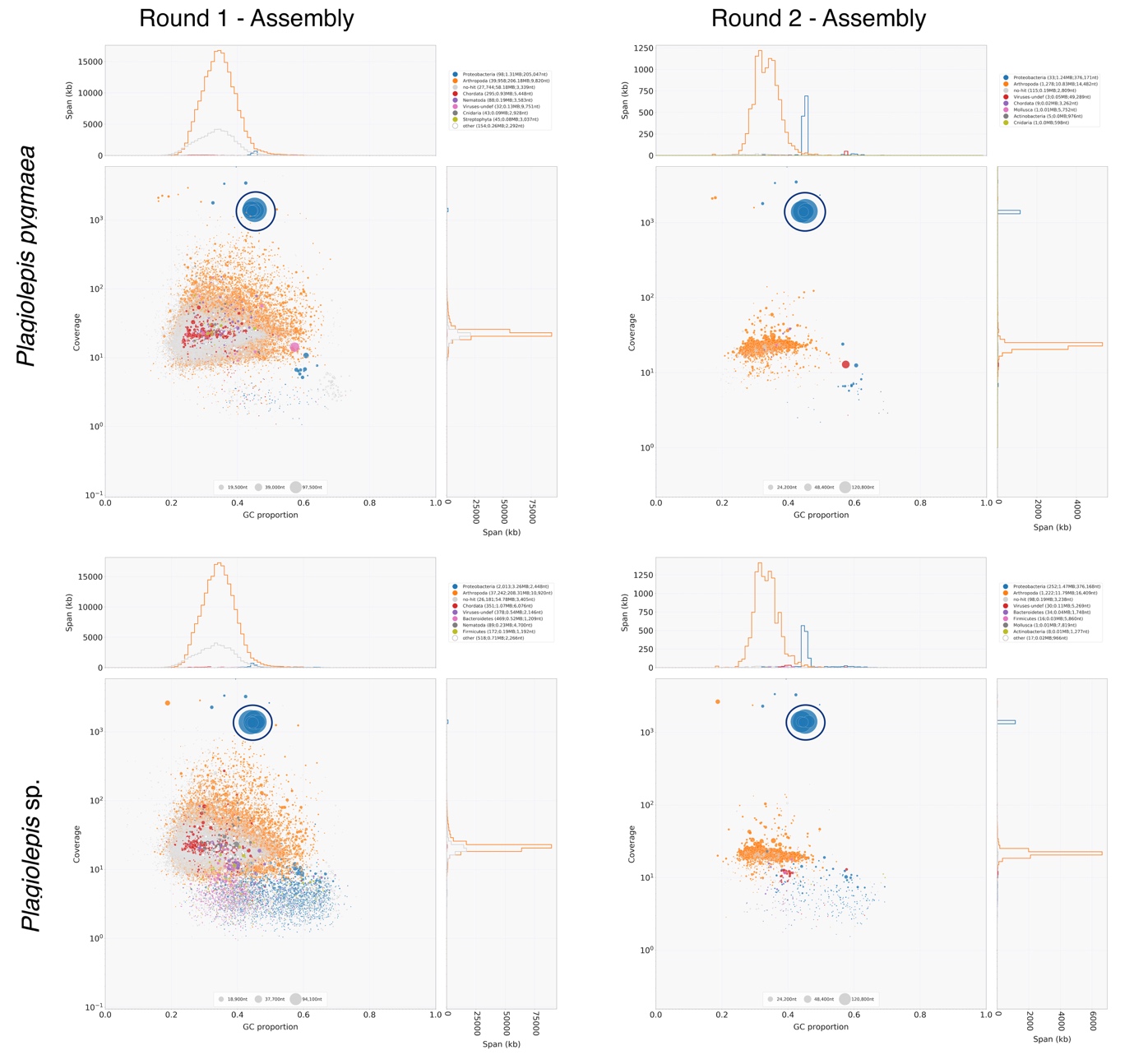
 **Figure S7:** Blobplots generated using BlobTools for both the first and second round of assembly for all metagenomic assemblies. Each dot represents a contig from the assembly plotted by GC proportion (x-axis) and coverage (y-axis). Dots are coloured by the taxonomic assignment at the level of phylum. Contigs taxonomically assigned to the symbiont at the level of species are indicated with a large dark blue circle.

**
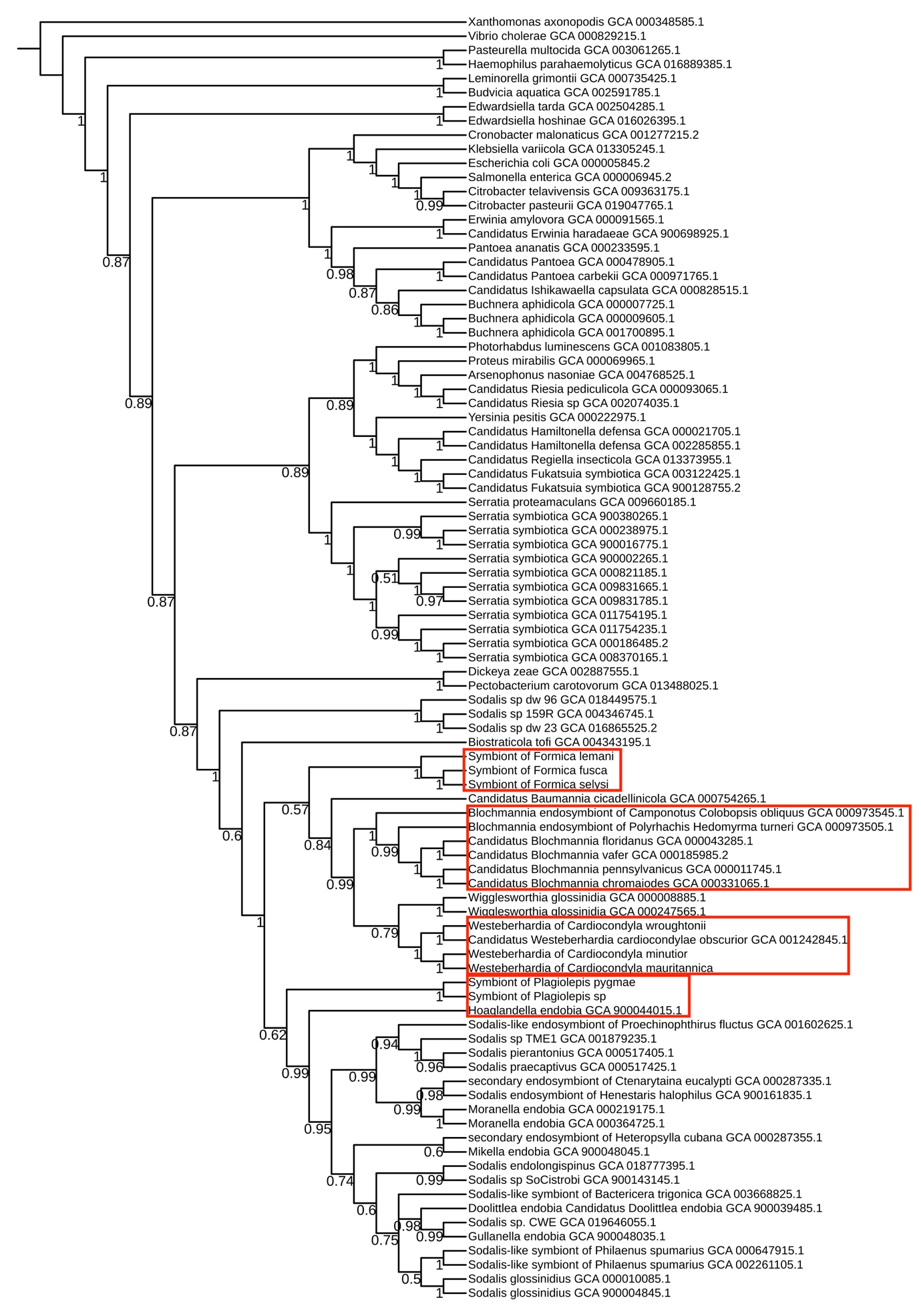
**

**Figure S8:** Full phylogeny of gammaproteobacterial endosymbionts and close free-living relatives constructed using phylobayes. Based on a dayhoff6 recoded amino acid alignment of 72 core gammaproteobacterial genes. Red boxes indicate ant symbionts of interest.


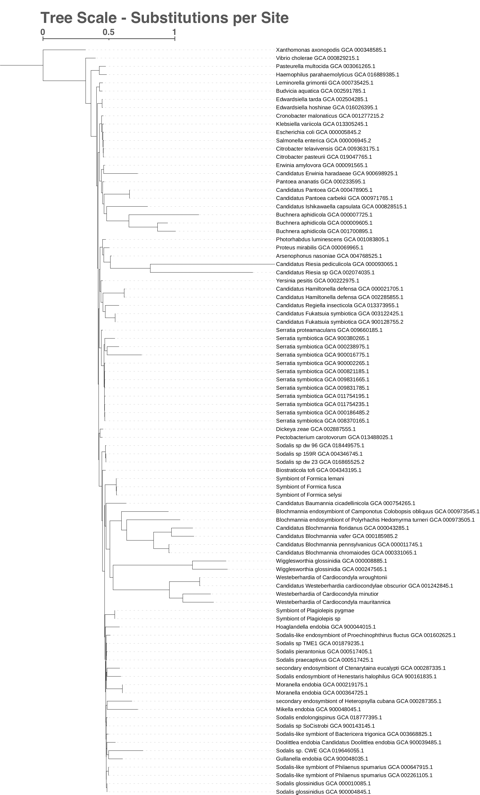


**Figure S9 :** Full phylogeny of gammaproteobacterial endosymbionts and close free-living relatives constructed using phylobayes, including branch lengths. Based on a dayhoff6 recoded amino acid alignment of 72 core gammaproteobacterial genes.


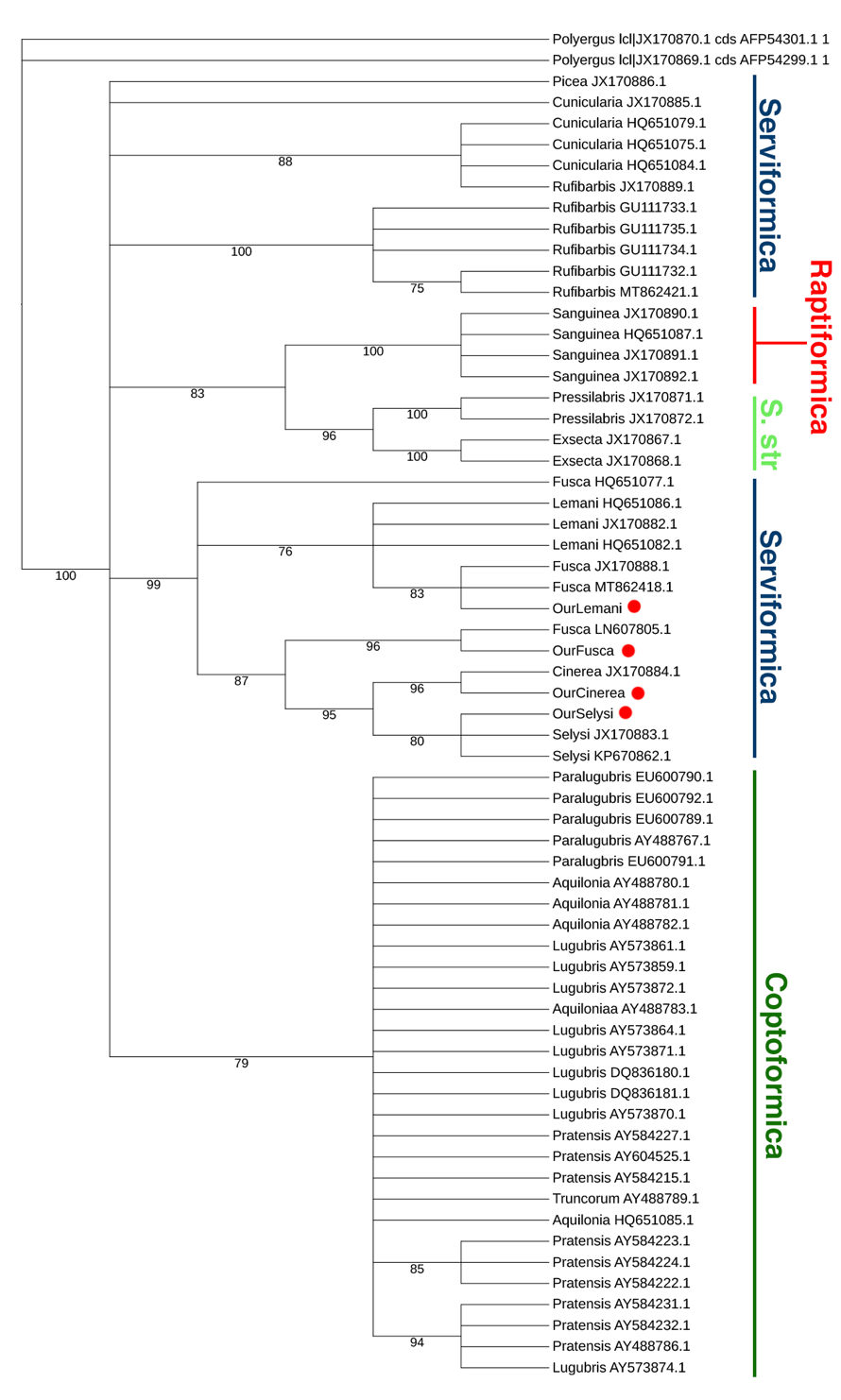


**Figure S10:** Phylogeny of *Formica* species based on cytochrome B sequences based on the work of (33). Sequences indicated with a red dot are sequences which came from individuals on which we preformed WGS. The phylogeny was created using PhyML (21) under default parameters with a bootstrap of 100.

**
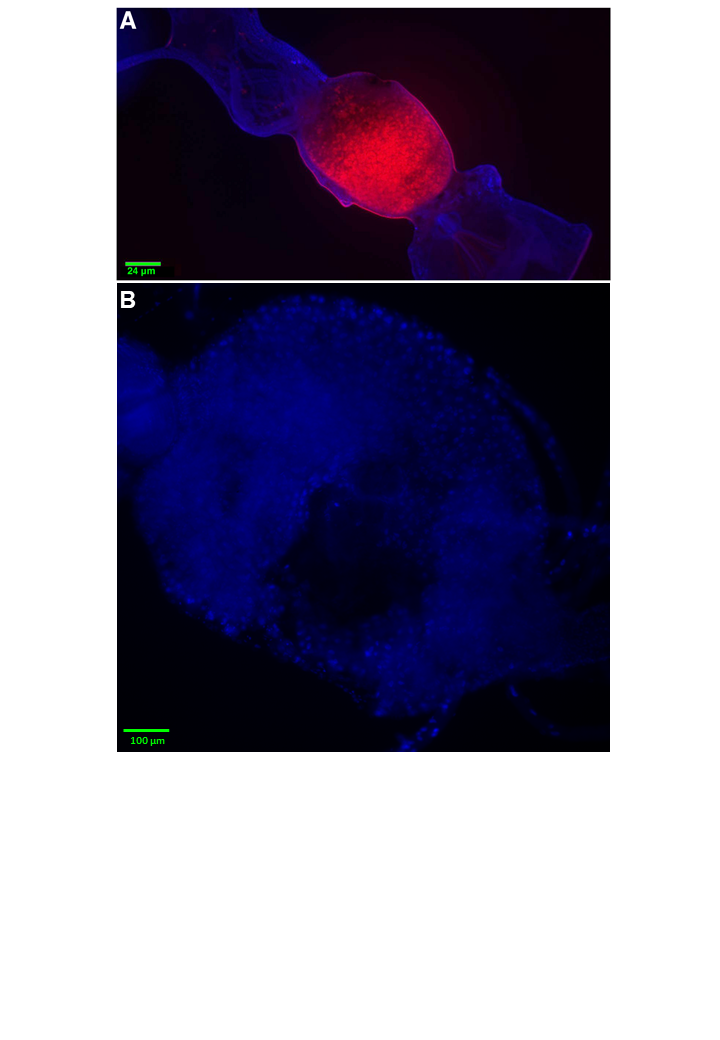
**

**Figure S11:** Fluorescent *in situ* hybridisation generated image of *Formica* spp. midguts. A. FISH on a *Formica fusca* queen midgut. DAPI staining in blue, symbiont stained in red. B. FISH on a *Formica lemani* queen midgut. DAPI staining in blue. The absence of red signal shows the absence of the symbiont.

**Datasets:**

All six of these tables are contained in a single excel file under the appropriately labelled tabs.

**S1:** A table showing the results of the gene enrichment analysis conducted using David as described in the supplementary methods section “Intergenomic Comparisons”.

**S2:** A table documenting the presence and absence of genes, categorised by synthetic pathway, in the genomes of *Blochmannia*, *Westeberhardia*, *Candidatus* Liliensternia, and *Candidatus* Jungenella symbionts as described in the supplementary methods section “Genome Assembly, Metagenome Investigation, Gene and Functional Annotation”.

**S3:** A table listing all samples subjected to diagnostic PCR screening, deep coverage 16S rRNA sequencing, or ITS sequencing along with relevant metadata as described in supplementary methods sections “Diagnostic PCR and Sanger Sequencing” and “16S rRNA and ITS Sequencing”.

**S4:** A table listing all samples we sent for whole genome sequencing along with relevant metadata and statistics on the endosymbiont genome assemblies derived from each sequencing run. The procedure is described in supplementary methods section “Whole Genome Sequencing” and “Genome Assembly, Metagenome Investigation, Gene and Functional Annotation”

**S5:** A table providing a breakdown of positive and negative queens and workers by species and colony based on the data provided in Dataset S3.

**S6:** A table listing all primers used in 16S rRNA, ITS, and Sanger sequencing as described in supplementary methods sections “Diagnostic PCR and Sanger Sequencing” and “16S rRNA and ITS Sequencing”.

**S7:** A table listing the fractional relative abundance of each OTU found at over 1% relative abundance in *Formica* samples.

**S8:** A table listing the taxonomic identifications assigned to each OTU listed in Table S7.

**S9:** A table listing the fractional relative abundance of each OTU found at over 1% relative abundance in *Cardiocondyla* samples.

**S10:** A table listing the taxonomic identifications assigned to each OTU listed in Table S9.

**SI References**

1. Bolger AM, Lohse M, Usadel B. Trimmomatic: A flexible trimmer for Illumina sequence data. Bioinformatics. 2014;30(15):2114–20.

2. Nurk S, Bankevich A, Antipov D, Gurevich A, Korobeynikov A, Lapidus A, et al. Assembling Genomes and Mini-metagenomes from Highly Chimeric Reads. In: Deng M, Jiang R, Sun F, Zhang X, editors. Research in Computational Molecular Biology. Berlin, Heidelberg: Springer Berlin Heidelberg; 2013. p. 158–70.

3. Li H, Durbin R. Fast and accurate short read alignment with Burrows-Wheeler transform. Bioinformatics. 2009;25:1754–60.

4. Laetsch DR, Blaxter ML, Leggett RM. BlobTools : Interrogation of genome assemblies. F1000Research 2017. 2017;6(1287):1–16.

5. Buchfink B, Xie C, Huson DH. Fast and sensitive protein alignment using DIAMOND. Nat Methods. 2015;12(1):59–60.

6. Camacho C, Coulouris G, Avagyan V, Ma N, Papadopoulos J, Bealer K, et al. BLAST+: architecture and applications. BMC Bioinformatics [Internet]. 2009;10:421. Available from: http://www.ncbi.nlm.nih.gov/pubmed/20003500%0Ahttp://www.pubmedcentral.nih.gov/articlerender.fcgi?artid=PMC2803857

7. Li H, Handsaker B, Wysoker A, Fennell T, Ruan J, Homer N, et al. The Sequence Alignment/Map format and SAMtools. Bioinformatics. 2009;25(16):2078–9.

8. Seemann T. Prokka: Rapid prokaryotic genome annotation. Bioinformatics. 2014;30(14):2068–9.

9. Tanizawa Y, Fujisawa T, Nakamura Y. DFAST: A flexible prokaryotic genome annotation pipeline for faster genome publication. Bioinformatics. 2018;34(6):1037–9.

10. Hyatt D, Chen GL, LoCascio PF, Land ML, Larimer FW, Hauser LJ. Prodigal: Prokaryotic gene recognition and translation initiation site identification. BMC Bioinformatics. 2010;11(119).

11. Riley M, Abe T, Arnaud MB, Berlyn MKB, Blattner FR, Chaudhuri RR, et al. Escherichia coli K-12: A cooperatively developed annotation snapshot - 2005. Nucleic Acids Res. 2006;34(1):1–9.

12. Tatusova T, DiCuccio M, Badretdin A, Chetvernin V, Nawrocki EP, Zaslavsky L, et al. NCBI prokaryotic genome annotation pipeline. Nucleic Acids Res [Internet]. 2016 Aug 19;44(14):6614–24. Available from: https://academic.oup.com/nar/article-lookup/doi/10.1093/nar/gkw569

13. Caspi R, Foerster H, Fulcher CA, Kaipa P, Krummenacker M, Latendresse M, et al. The MetaCyc Database of metabolic pathways and enzymes and the BioCyc collection of pathway/genome databases. Nucleic Acids Res. 2008;36(SUPPL. 1):623–31.

14. Dale C, Young SA, Haydon DT, Welburn SC. The insect endosymbiont Sodalis glossinidius utilizes a type III secretion system for cell invasion. Proc Natl Acad Sci [Internet]. 2001 Feb 13;98(4):1883–8. Available from: http://www.pnas.org/cgi/doi/10.1073/pnas.98.4.1883

15. Santos-Garcia D, Rollat-Farnier PA, Beitia F, Zchori-Fein E, Vavre F, Mouton L, et al. The genome of cardinium cBtQ1 provides insights into enome reduction, symbiontmotility, and its settlement n bemisia tabaci. Genome Biol Evol. 2014;6(4):1013–30.

16. Emms DM, Kelly S. OrthoFinder: solving fundamental biases in whole genome comparisons dramatically improves orthogroup inference accuracy. Genome Biol [Internet]. 2015;16(1):1–14. Available from: http://dx.doi.org/10.1186/s13059-015-0721-2

17. Husnik F, McCutcheon JP. Repeated replacement of an intrabacterial symbiont in the tripartite nested mealybug symbiosis. Proc Natl Acad Sci [Internet]. 2016 Sep 13;113(37):E5416–24. Available from: http://www.pnas.org/lookup/doi/10.1073/pnas.1603910113

18. Huang DW, Sherman BT, Lempicki RA. Bioinformatics enrichment tools: Paths toward the comprehensive functional analysis of large gene lists. Nucleic Acids Res. 2009;37(1):1–13.

19. Huang DW, Sherman BT, Lempicki RA. Systematic and integrative analysis of large gene lists using DAVID bioinformatics resources. Nat Protoc [Internet]. 2009 Jan 18;4(1):44–57. Available from: http://www.nature.com/articles/nprot.2008.211

20. Edgar RC. MUSCLE: Multiple sequence alignment with high accuracy and high throughput. Nucleic Acids Res. 2004;32(5):1792–7.

21. Guindon S, Dufayard J, Lefort V. New Algorithms and Methods to Estimate Maximim-Likelihood Phylogenies Assessing the Performance of PhyML 3.0. Syst Biol. 2010;59(3):307–21.

22. Koga R, Tsuchida T, Fukatsu T. Quenching autofluorescence of insect tissues for in situ detection of endosymbionts. Appl Entomol Zool. 2009;

23. Sanders JG, Lukasik P, Frederickson ME, Russell JA, Koga R, Knight R, et al. Dramatic differences in gut bacterial densities correlate with diet and habitat in rainforest ants. Integr Comp Biol. 2017;57(4):705–22.

24. Simon C, Frati F, Beckenbach A, Crespi B, Liu H, Flook P. Evolution, Weighting, and Phylogenetic Utility of Mitochondrial Gene Sequences and a Compilation of Conserved Polymerase Chain Reaction Primers. Ann Entomol Soc Am [Internet]. 1994 Nov 1;87(6):651–701. Available from: https://academic.oup.com/aesa/article-lookup/doi/10.1093/aesa/87.6.651

25. Villesen P, Mueller UG, Schultz TR, Adams RMM, Bouck AC. EVOLUTION OF ANT-CULTIVAR SPECIALIZATION AND CULTIVAR SWITCHING IN APTEROSTIGMA FUNGUS-GROWING ANTS. Evolution (N Y) [Internet]. 2004 Oct;58(10):2252–65. Available from: http://doi.wiley.com/10.1111/j.0014-3820.2004.tb01601.x

26. Caporaso JG, Lauber CL, Walters WA, Berg-Lyons D, Lozupone CA, Turnbaugh PJ, et al. Global patterns of 16S rRNA diversity at a depth of millions of sequences per sample. Proc Natl Acad Sci U S A. 2011;108(SUPPL. 1):4516–22.

27. Epp LS, Boessenkool S, Bellemain EP, Haile J, Esposito A, Riaz T, et al. New environmental metabarcodes for analysing soil DNA: Potential for studying past and present ecosystems. Mol Ecol. 2012;21(8):1821–33.

28. P. D. Schloss, S. L. Westcott, T. Ryabin, J. R. Hall, M. Hartmann, E. B. Hollister, et al. Introducing mothur: Open-Source, Platform-Independent, Community-Supported Software for Describing and Comparing Microbial Communities. Appl Environ Microbiol. 2009;75(23):7537–41.

29. Wickham H. ggplot2: Elegant Graphics for Data Analysis [Internet]. Springer-Verlag New York; 2016. Available from: https://ggplot2.tidyverse.org

30. Edgar RC. Search and clustering orders of magnitude faster than BLAST. Bioinformatics [Internet]. 2010 Oct 1;26(19):2460–1. Available from: https://academic.oup.com/bioinformatics/article-lookup/doi/10.1093/bioinformatics/btq461

31. Edgar RC. UPARSE: highly accurate OTU sequences from microbial amplicon reads. Nat Methods [Internet]. 2013;10(10):996–8. Available from: http://www.ncbi.nlm.nih.gov/pubmed/23955772

32. Cole JR, Wang Q, Fish JA, Chai B, McGarrell DM, Sun Y, et al. Ribosomal Database Project: data and tools for high throughput rRNA analysis. Nucleic Acids Res [Internet]. 2014 Jan;42(D1):D633–42. Available from: https://academic.oup.com/nar/article-lookup/doi/10.1093/nar/gkt1244

33. Goropashnaya A V., Fedorov VB, Seifert B, Pamilo P. Phylogenetic relationships of Palaearctic Formica species (hymenoptera, Formicidae) based on mitochondrial cytochrome b sequences. PLoS One. 2012;7(7).
